# Supplementary material for: Fate Mapping Quantifies the Dynamics of B Cell Development and Activation throughout Life
Source: Cell Rep. Author manuscript; Available in PMC 2021 Nov 26. (PMC8622872; doi:10.1016/j.celrep.2020.108376)
Supplement: 1 [file NIHMS1647629-supplement-1.pdf]

Cell Reports, Volume *33*

Supplemental Information

Fate Mapping Quantifies the Dynamics of B Cell Development and Activation throughout Life

Melissa Verheijen, Sanket Rane, Claire Pearson, Andrew J. Yates, and Benedict Seddon

Supplemental Information

Figure S1: Variation in the degree of stable chimerism in B cell subsets within the same mice. Related to Figure 1.

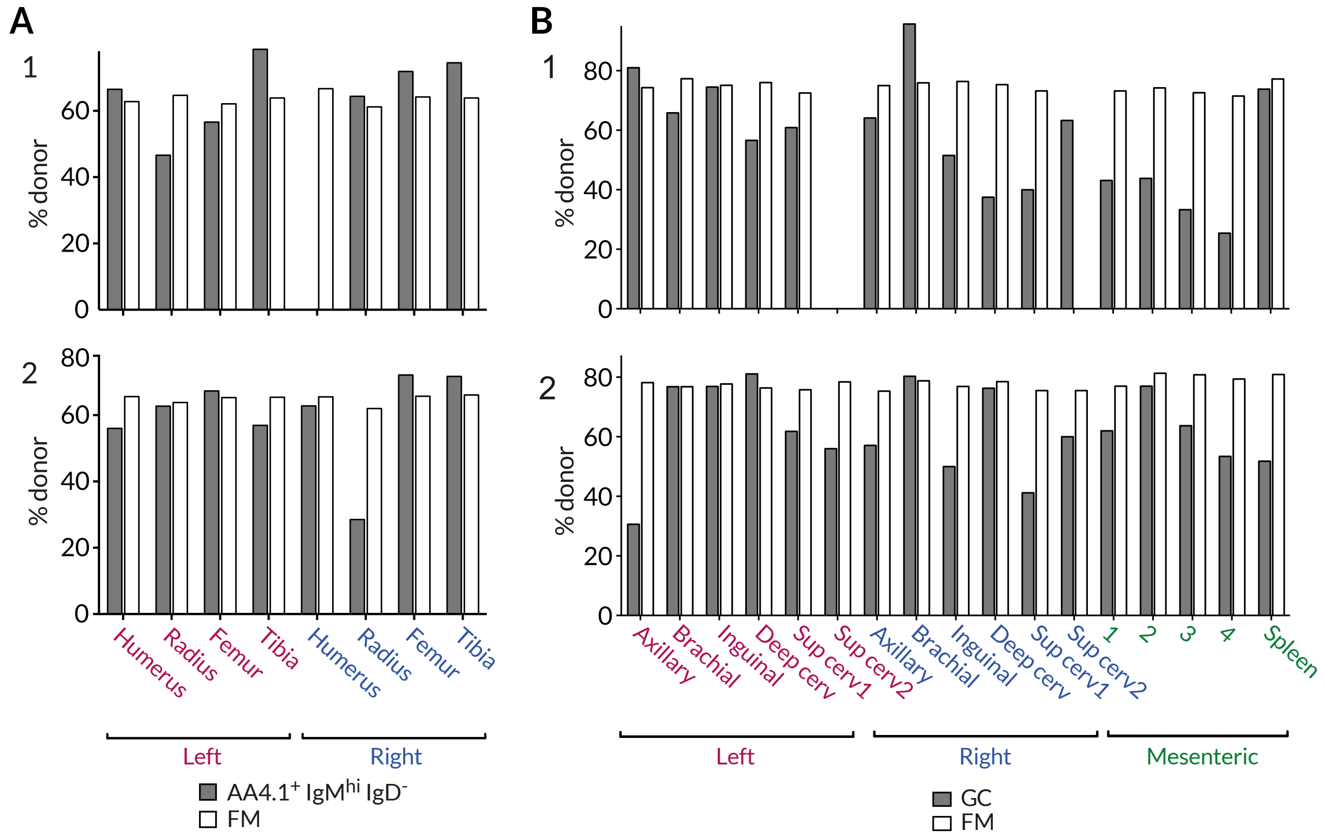

(A) Variation in the chimerism among AA4.1^{+}IgM^{hi}IgD^{-} B cell progenitors and recirculating FM B cells within different BM sites. **(B)** Variation in chimerism across different lymph nodes. Data from two representative animals.

Figure S2: Empirical descriptions of the timecourses of numbers (A-D) and chimerism (E-H) of potential B cell precursors of FM and GC B cells. Related to Figure 4.

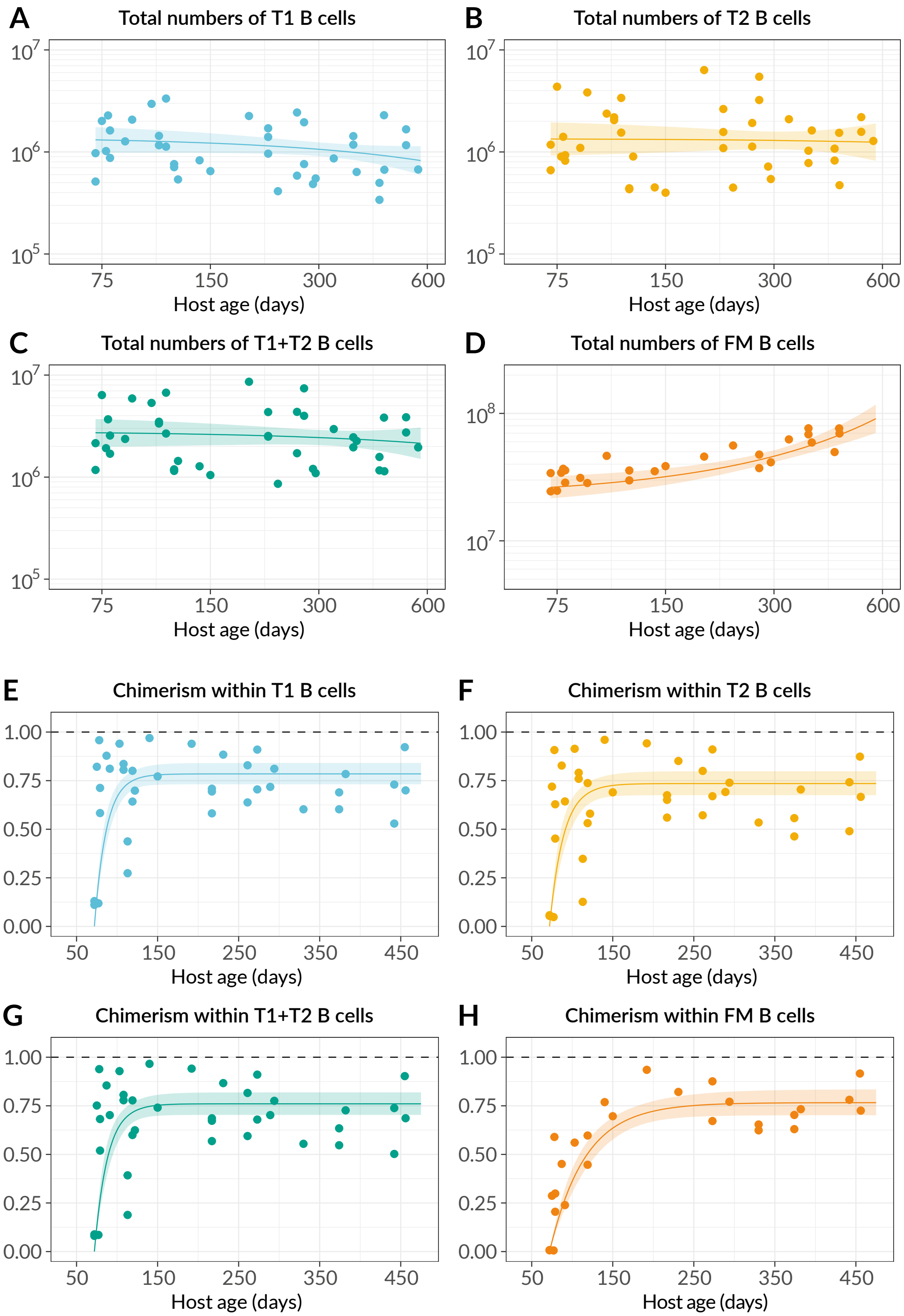

Figure S3: Fitted alternative models of FM B cell dynamics. Related to Figure 4.

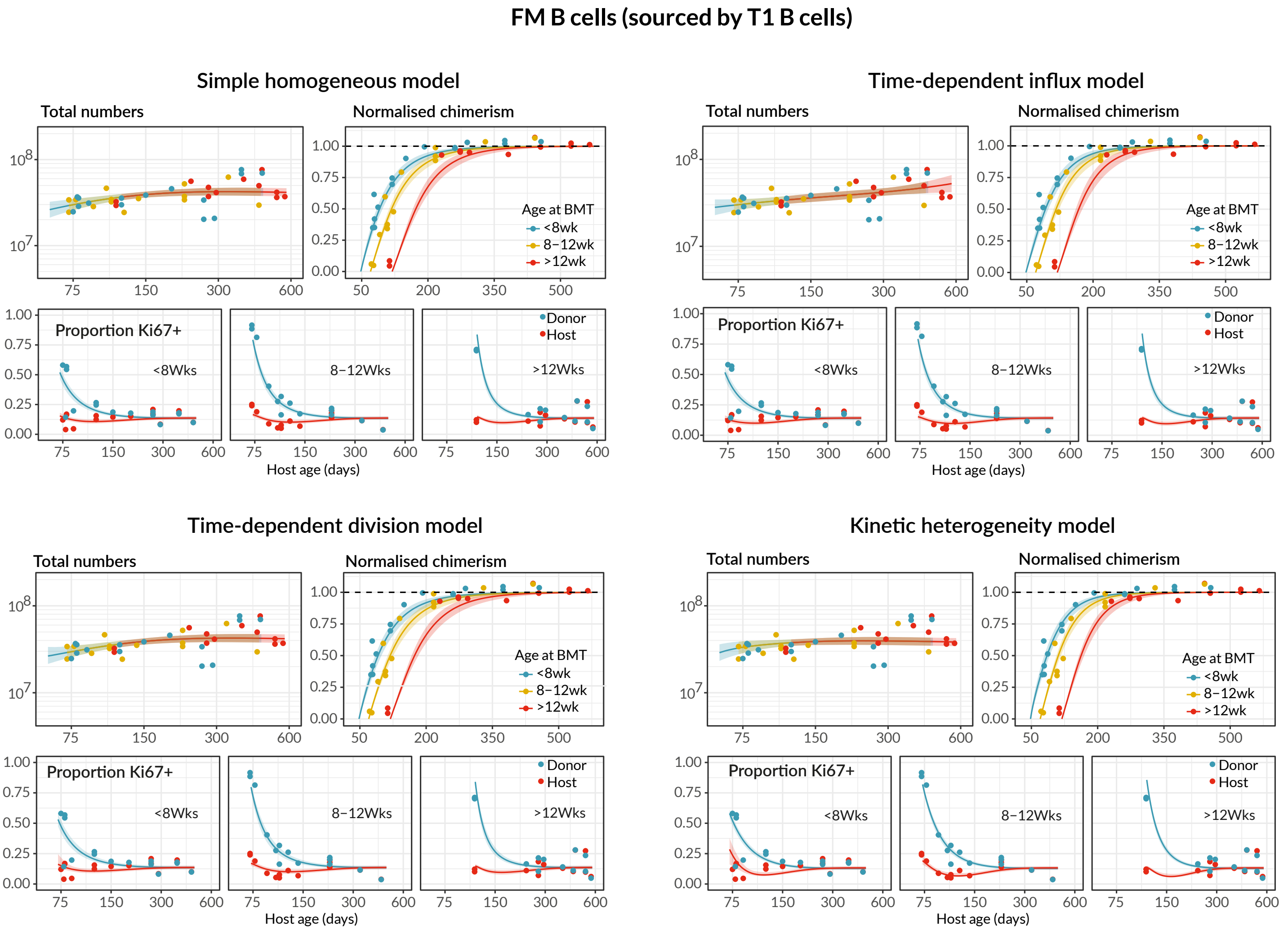

Figure S4: Predictions from competing models of FM B cell dynamics in young mice. Related to Figure 5.

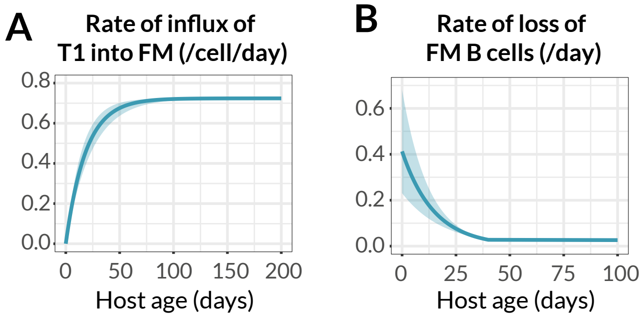

(**A**) Best fitting functional form for the change in *per capita* daily rate of influx from T1 to FM. (**B**) Best fitting functional form for the change in loss rate of FM with age.

Figure S5: Comparing germinal centre B cell numbers in germ free, busulfan chimeric and WT mice. Related to Figure 6.

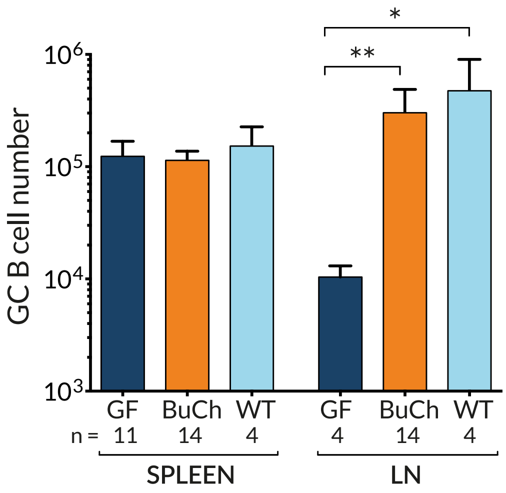

Absolute numbers of GC B cells in spleen and total LN from germ free (GF), busulfan chimeras (BuCh), and WT controls from a conventional barrier facility. Groups were of ages 10 weeks, 10-20 weeks and 10-14 weeks respectively. * $p<0.05$, ** $p<0.01$.

Figure S6: Fitted alternative models of splenic germinal centre B cell dynamics. Related to Figure 6.

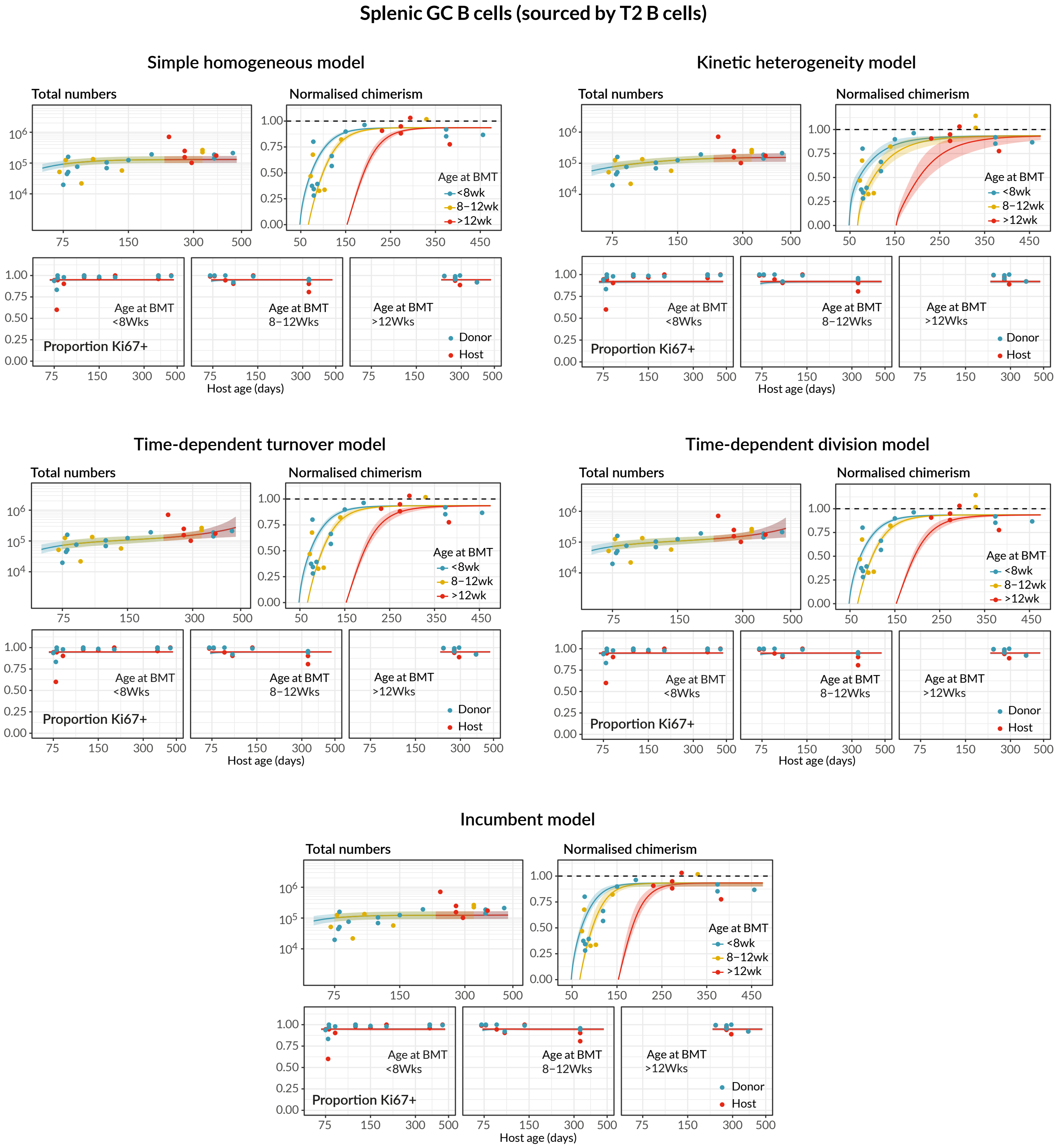

Figure S7: Fitted alternative models of lymph node germinal centre B cell dynamics. Related to Figure 6.

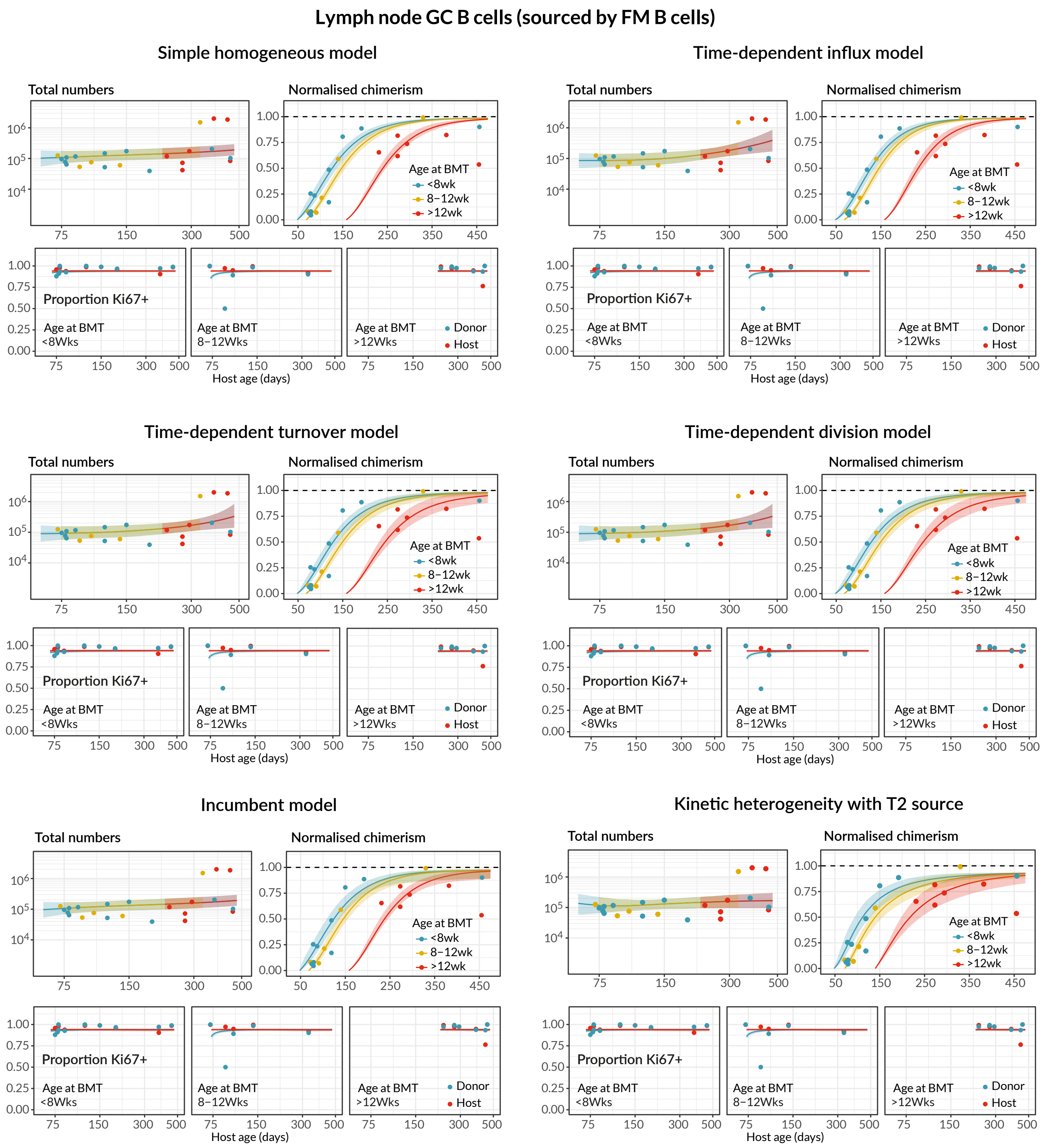

Figure S8: Age-associated B cells (AABC) accumulate in older mice. Related to Figure 4.

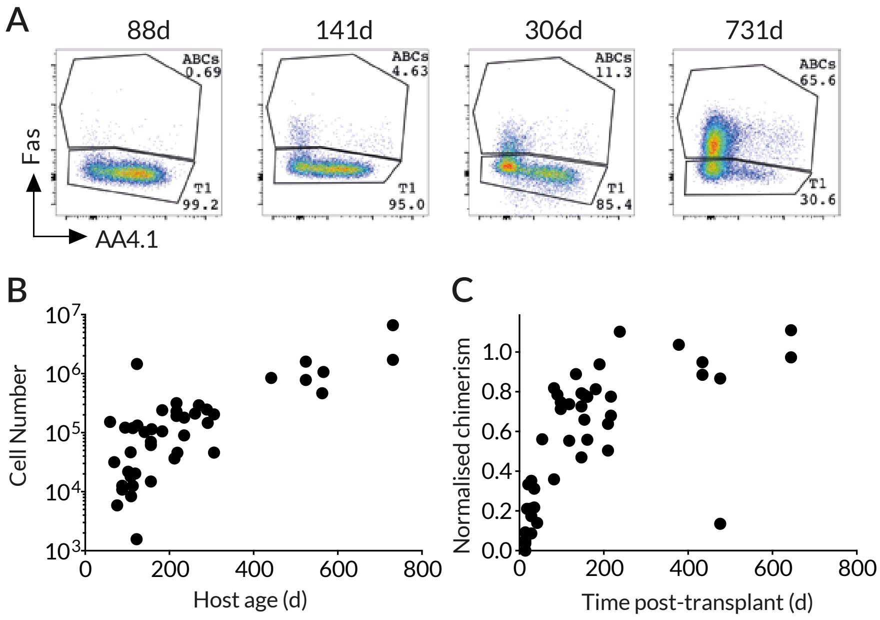

(A) Gating strategy for AABC. **(B)** AABC increase in number with host age. **(C)** AABC turn over within ∼300 days.

Table S1: Comparison of models describing the population dynamics of Follicular Mature (FM) B cells, pooled from LN and spleen, using Akaike weights (Methods S1, part D) as percentage measures of relative support. The most strongly favoured model is highlighted. Related to Figure 4.

Source	Model and Akaike weight (%)				
	Simple homogeneous	Time-dependent turnover	Time-dependent division	Time-dependent influx	Kinetic heterogeneity
**T1**	9.0	**64**	3.0	18	5.0
**T2**	0.0	0.0	0.0	0.0	0.0
**T1 + T2**	0.0	0.5	0.0	0.5	0.0

Table S2: Comparison of models describing the population dynamics of germinal centre (GC) B cells in spleen, with or without the additional information regarding proliferation from the Ki67 reporter mice. *Showed weak time-dependence in the rates of turnover (halving every ∼800 mo) or division (doubling every >1000 mo); were rejected in favour of the simple homogeneous model. Related to Figure 6.

Source	Model and Akaike weight (%)					
	Simple homogeneous	Time-dependent turnover	Time-dependent division	Time-dependent influx	Kinetic heterogeneity	Incumbent
Without informing models using data from Ki67-Cre-ER^{T2}-YFP reporter mice						
T1	18	16	7	12	5	18
T2	5	11	1	2	1	4
Informing models using data from Ki67-Cre-ER^{T2}-YFP reporter mice						
T1	0	3	2	9	0	0
T2	2	16*	11*	**57**	0	0

Table S3: Comparison of models describing the population dynamics of GC B cells in lymph nodes. Related to Figure 6.

Source	Model and Akaike weight (%)					
	Simple homogeneous	Time-dependent influx	Time-dependent turnover	Time-dependent division	Kinetic heterogeneity	Incumbent
Without informing models using data from Ki67-Cre-ER^{T2}-YFP reporter mice						
T1	0	21	1	0	0	0
T2	0	22	0	0	0	0
FM	6	37	11	2	0	0
Informing models using data from Ki67-Cre-ER^{T2}-YFP reporter mice						
T1	0	0	0	0	5	0
T2	0	0	0	0	11	0
FM	0	0	0	0	**84**	0

Methods S1

Details of all mathematical and statistical analyses; related to Figures 4-6.

A – Modelling the dynamics of precursor cell numbers and chimerism

We considered T1, T2, and T1+T2 combined as the potential direct precursors (sources) of FM B cells, and T1, T2 and FM B cells as potential sources of GC B cells. In adult mice, we described the time-variation in the sizes of these populations with the empirical descriptor function $S(t) = S_0\, e^{-\nu\, t}$ (Fig. **??**, panels A-D), where the parameters S_0 and ν were estimated by fitting to the log-transformed cell counts using least squares.

Similarly, the timecourses of donor chimerism in these populations were all described well with $\chi(t) = \chi_{\text{stable}}\,(1 - e^{-\nu\, t})$, shown in Fig. **??**, panels E-H; here, χ_{stable} and ν were estimated using non-linear least squares.

We assumed a constant *per capita* rate of influx ψ from the source $S(t)$, giving a total influx of $\phi(t) = \psi S(t)$ cells/day. The daily influx of host and donor cells into the target population is then

$$\begin{aligned} \phi_{\text{donor}}(t) &= \psi\, S(t)\, \chi(t), \\ \phi_{\text{host}}(t) &= \phi(t) - \phi_{\text{donor}}(t). \end{aligned} \tag{S1}$$

The unknown ψ is estimated along with the other model parameters. In the time-dependent recruitment model, we assumed the form $\psi(t) = \psi_0\, e^{p\, t}$, and estimated ψ_0 and p.

B – Mathematical models

Simple homogeneous model: In this model we assume that cells form a kinetically homogeneous population that self-renews through homeostatic division with first-order kinetics at rate α, and is lost (turns over) at a rate δ, which combines death and onward differentiation. The inverse of α is the mean interdivision time, and the inverse of δ is the mean residence time of a cell. Influx of cells from the source compartment is denoted $\phi(t)$, which is the product of the *per capita* rate of influx ψ and the timecourse of the size of precursor population $S(t)$, which is described empirically (see part A). We model the dynamics of Ki67^{hi} (H) and Ki67^{lo} (L) cells using the following ODE model;

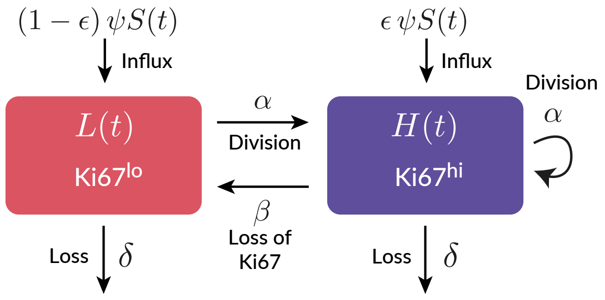

$$\begin{aligned} \dot{L}(t) &= \psi S(t)(1-\epsilon)\; + \beta\, H(t) - (\alpha + \delta)\, L(t), \\ \dot{H}(t) &= \psi S(t)\epsilon\; + \alpha\,(2\, L(t) + H(t)) - (\beta + \delta)\, H(t). \end{aligned} \tag{S2}$$

Here, β is the rate of loss of Ki67 expression after mitosis, and ϵ is the proportion of the cells entering from the source that are Ki67^{hi}; we used its average observed value in the model fitting process. We assumed eqns. S2 held identically for host and donor cells. We fitted the following combinations of the solutions to these equations simultaneously to

the timecourses of

$$\begin{aligned}
\text{Total cell numbers} &= N(t) = H_{\text{donor}}(t) + H_{\text{host}}(t) + L_{\text{donor}}(t) + L_{\text{host}}(t) \\
\text{Normalised chimerism} &= \frac{1}{\chi_{\text{source}}(t)} \frac{H_{\text{donor}}(t) + L_{\text{donor}}(t)}{N(t)} \\
\text{Proportions of donor and host cells expressing Ki67} &= \frac{H_{\text{donor}}(t)}{H_{\text{donor}}(t) + L_{\text{donor}}(t)}, \quad \frac{H_{\text{host}}(t)}{H_{\text{host}}(t) + L_{\text{host}}(t)},
\end{aligned}$$

using the empirical descriptions of the size ($S(t)$) and chimerism ($\chi_{\text{source}}(t)$) of the source population (see part A). We define time t_0 to be age at BMT of the youngest recipient (approximately 7 weeks), at which time the size of donor compartment is zero. Therefore, the Ki67^{hi} proportion among donor cells at t_0 reflects that in the source, $\kappa_{\text{donor}}(t_0) = \epsilon$. The Ki67^{hi} proportion among host cells at t_0 is defined as κ_0. We estimated β, α, δ, ψ, κ_0 and $H(t_0) + L(t_0)$, the size of the host compartment at t_0.

Time-dependent models: In these extensions of the model above, the *per capita* rate of influx of new cells from the source population ψ, the rate of cell division α, or the rate of loss δ may vary with time. We allowed each sub-model to exhibit time-dependence in only one process. These three sub-models are also homogeneous; at any given instant, all cells in the population exhibit the same rates of division and turnover. We assumed that the time-dependent parameter varied with mouse age t as $\exp(rt)$, where r was estimated from the data and was unconstrained (*i.e.* the rate constant could either rise or fall with time).

Kinetic-heterogeneity model: This model comprises two subsets, which are independent, fed separately from the same source population, and are lost and/or divide at different rates. The resulting dynamics of the population as a whole are therefore the weighted average of the more 'transient' subset (rapid net loss, $\delta_f - \alpha_f$) and a 'persistent' subset (slower net loss, $\delta_s - \alpha_s$). We solve the following equations for Ki67^{hi} and Ki67^{lo} cells among the transient and persistent subsets, and formulate it identically for host and donor cells;

$$\begin{aligned}
\dot{H}_f(t) &= \phi(\psi, t)\, f\, \epsilon \; + \alpha_f \,(2\, L_f(t) + H_f(t)) - (\beta + \delta_f)\, H_f(t), \\
\dot{L}_f(t) &= \phi(\psi, t)\, f\, (1 - \epsilon) \; + \beta\, H_f(t) - (\alpha_f + \delta_f)\, L_f(t), \\
\dot{H}_s(t) &= \phi(\psi, t)\, (1 - f)\, \epsilon \; + \alpha_s \,(2\, L_s(t) + H_s(t)) - (\beta + \delta_s)\, H_s(t), \\
\dot{L}_s(t) &= \phi(\psi, t)\, (1 - f)\, (1 - \epsilon) \; + \beta\, H_s(t) - (\alpha_s + \delta_s)\, L_s(t).
\end{aligned} \tag{S3}$$

Along with the kinetic parameters we also estimate the proportions of the transient subset in the precursor (f) and in the target (q) populations, and the initial fractions of Ki67^{hi} cells in the transient and persistent subsets. The initial numbers of host-derived cells in the transient and persistent subsets are defined as $N_0\, q$ and $N_0\,(1 - q)$, respectively.

Incumbent model: In this model, described in Hogan et al. (2015) and Rane et al. (2018), heterogeneity is exhibited only in the host compartment, which is assumed to comprise (i) an 'incumbent' subset of older, self-renewing cells that are resistant to displacement by new cells and (ii) a 'displaceable' subset that is replaced continuously by cohorts of new cells entering the pool. All donor cells are assumed to behave as displaceable cells.

$$\begin{aligned}
\dot{H}(t) &= \phi(\psi, t)\epsilon \; + \alpha\,(2\, L(t) + H(t)) - (\beta + \delta)\, H(t), \\
\dot{L}(t) &= \phi(\psi, t)(1 - \epsilon) \; + \beta\, H(t) - (\alpha + \delta)\, L(t), \\
\dot{H}_{\text{inc}}(t) &= \alpha_{\text{inc}}\,(2\, L_{\text{inc}}(t) + Y_{\text{inc}}(t)) - (\beta + \delta_{\text{inc}})\, H_{\text{inc}}(t), \\
\dot{L}_{\text{inc}}(t) &= \beta\, H_{\text{inc}}(t) - (\alpha_{\text{inc}} + \delta_{\text{inc}})\, L_{\text{inc}}(t).
\end{aligned} \tag{S4}$$

We assume that the incumbent subset is established early in life, before the minimum age of BMT in our chimeric animals (∼7 weeks).

C – The time taken to approach to stable chimerism in a B cell population is determined predominantly by the clonal lifetime

Here we illustrate for the simplest homogenous model the factors that determine the rate at which chimerism in a population reaches that of its precursor population. Assume a population $N(t)$ is fed by precursors at constant total rate ϕ, divides at *per capita* rate α and is lost through death or differentiation at *per capita* rate δ;

$$dN/dt = \phi - (\delta - \alpha)N(t).$$

The quantity $\delta - \alpha$ is the net loss rate, which we denote λ:

$$dN/dt = \phi - \lambda N(t).$$

Assume the source acquires a stable chimerism χ, and that host (h) and donor (d) cells behave identically;

$$\begin{aligned} dN_d/dt &= \chi\phi - \lambda N_d(t) \\ dN_h/dt &= (1-\chi)\phi - \lambda N_h(t) \end{aligned}$$

The normalised chimerism of the population is

$$\chi_{\text{norm}} = \frac{N_d}{\chi(N_d + N_h)},$$

which evolves according to

$$\begin{aligned} \frac{d}{dt}\chi_{\text{norm}}(t) &= \frac{1}{\chi N(t)}\left(\chi\phi - \lambda N_d(t) - \frac{N_d(t)}{N(t)}\frac{dN(t)}{dt}\right) \\ &= \frac{1}{\chi N(t)}\left(\chi\phi - \lambda N_d(t) - \frac{N_d(t)}{N(t)}\left(\phi - \lambda N(t)\right)\right) \\ &= \frac{1}{\chi N(t)}\left(\chi\phi - \phi\frac{N_d(t)}{N(t)}\right) \\ &= \frac{\phi}{N(t)}\Big(1 - \chi_{\text{norm}}(t)\Big). \end{aligned} \tag{S5}$$

If the population is at equilibrium then $N(t) = \phi/\lambda$, giving

$$\frac{d}{dt}\chi_{\text{norm}}(t) = \lambda\Big(1 - \chi_{\text{norm}}(t)\Big), \tag{S6}$$

which implies

$$\chi_{\text{norm}}(t) = 1 - e^{-\lambda t}; \tag{S7}$$

that is, the chimerism in the population reaches that of its precursors at a rate determined purely by the clonal lifespan $1/\lambda$. If the population is initially out of equilibrium at size N_0,

$$\chi_{\text{norm}}(t) = 1 - \frac{1}{1 + (e^{\lambda t} - 1)\frac{\phi}{\lambda N_0}}, \tag{S8}$$

the rate of approach to $\chi_{\text{norm}} = 1$ is then governed by both λ and the daily influx as a proportion of the initial pool size, ϕ/N_0 (intuitively, if the pool is initially over-populated, $\phi/\lambda N_0 < 1$ and chimerism increases more slowly because of the excess of host cells; if the pool is depleted, $\phi/\lambda N_0 > 1$ and stable chimerism is achieved more quickly). Equation S8 reduces to S7 when $N_0 = \phi/\lambda$.

D – Fitting and selecting mathematical models

Likelihood

We attempted to explain the kinetics of host and donor cells in busulfan chimeric mice with an array of mathematical models, detailed in the main text and illustrated in Fig. 4A. As described, variation in the degree of depletion of host HSCs by busulfan treatment led to mouse-to-mouse variation in the level of stable bone-marrow chimerism (the fraction that are donor-derived), and hence also in peripheral subsets. We removed this variation by dividing the chimerism in each B cell subset by the chimerism χ in the T1 precursor population. This normalised chimerism (donor fraction) is

$$\chi_{\text{norm}} = \frac{\text{Donor cell numbers}}{\text{Total cell numbers} \times \chi}.$$

This approach allows us to fit a single model to data from multiple mice. Each model was fitted simultaneously to the timecourses of total cell counts ($N(t)$, the sum of host and donor cells), the normalised chimerism $\chi_{\text{norm}}(t)$, and the proportions of Ki67^{hi}cells in the host and donor compartments ($\kappa_{\text{host}}(t)$ and $\kappa_{\text{donor}}(t)$). Cell counts were log-transformed while χ_{norm}, κ_{host} and κ_{donor} were logit-transformed, to ensure that measurement errors were approximately normally distributed. The joint likelihood of the datasets (with variables representing their transformed values) is then

$$\begin{aligned}\mathcal{L} = \prod_{i=1}^{n} & \frac{\exp\left(\frac{-(N_i - N_i^{\text{pred}})^2}{2\sigma_N^2}\right)}{\sqrt{2\pi}\sigma_N} \times \frac{\exp\left(\frac{-(\chi_{\text{norm},i} - \chi_{\text{norm},i}^{\text{pred}})^2}{2\sigma_\chi^2}\right)}{\sqrt{2\pi}\sigma_\chi} \times \frac{\exp\left(\frac{-(\kappa_{i,\text{host}} - \kappa_{i,\text{host}}^{\text{pred}})^2}{2\sigma_{\kappa_{\text{host}}}^2}\right)}{\sqrt{2\pi}\sigma_{\kappa,\text{host}}} \times \frac{\exp\left(\frac{-(\kappa_{i,\text{donor}} - \kappa_{i,\text{donor}}^{\text{pred}})^2}{2\sigma_{\kappa_{\text{donor}}}^2}\right)}{\sqrt{2\pi}\sigma_{\kappa,\text{donor}}} \\ \equiv & \frac{\exp(-\text{SSR}_N/2\sigma_N^2)}{\left(\sqrt{2\pi}\sigma_N\right)^n} \times \frac{\exp(-\text{SSR}_\chi/2\sigma_\chi^2)}{\left(\sqrt{2\pi}\sigma_\chi\right)^n} \times \frac{\exp(-\text{SSR}_{\kappa,\text{host}}/2\sigma_{\kappa,\text{host}}^2)}{\left(\sqrt{2\pi}\sigma_{\kappa,\text{host}}\right)^n} \times \frac{\exp(-\text{SSR}_{\kappa,\text{donor}}/2\sigma_{\kappa,\text{donor}}^2)}{\left(\sqrt{2\pi}\sigma_{\kappa,\text{donor}}\right)^n},\end{aligned}$$

where n is the number of animals, each yielding four observations, and SSR denotes the sum of squared residuals, with each being a function of the data and the model parameters. This gives the joint log-likelihood (up to a constant);

$$\log \mathcal{L} = -\frac{1}{2}\left(\frac{\text{SSR}_N}{\sigma_N^2} + \frac{\text{SSR}_\chi}{\sigma_\chi^2} + \frac{\text{SSR}_{\kappa,\text{host}}}{\sigma_{\kappa,\text{host}}^2} + \frac{\text{SSR}_{\kappa,\text{donor}}}{\sigma_{\kappa,\text{donor}}^2}\right) - n(\log\sigma_N + \log\sigma_\chi + \log\sigma_{\kappa,\text{host}} + \log\sigma_{\kappa,\text{donor}}).$$

Parameter estimation

We used a Bayesian approach to estimating the model parameters, the errors associated with the measurements in each dataset, and a measure of support for each model. The inputs to this procedure are the joint likelihood shown above, and a set of prior distributions on the model parameters and the unknown measurement errors in each dataset. We refer to these unknowns collectively as θ. The Bayesian procedure updates these priors with the likelihood, to generate posterior distributions of θ that reflect our knowledge of these parameters in the light of the data, collectively denoted y. Strong (narrow) priors help to regularise a model's behaviour and prevent it from learning too much from the data – and hence guard against over-fitting. The joint posterior distribution of the

parameters is calculated using Bayes' rule,

$$p(\theta_{\text{post}}|y) = \frac{p(y|\theta_{\text{post}}) \cdot p(\theta_{\text{prior}})}{p(y)} \tag{S9}$$

where $p(y)$ is the likelihood of the data (averaged over the priors) that normalises the posterior such that it integrates to 1. We consider priors to be tools that improve a model's ability to learn from the data, and subjected them to similar standards of evaluation and revaluation as any other component of the model. Detailed descriptions of the priors, together with the code and data for performing all of the analyses presented in this study, are available at `github.com/sanketrane/B_cells_FM_GC`.

The models were represented as systems of ordinary differential equations (ODEs), described in detail in Methods S1, part B. We solved them numerically using the *integrate_ode_rk45* solver in the *Stan* programming language and used the default no-U-turn sampler (NUTS) to generate the posterior distributions of the parameters. We confirmed that the log-transformed cell counts, and the logit-transformed values of the normalised chimerism and the Ki67^{hi} proportions in host and donor compartments, were all normally distributed with constant errors (standard deviations). These standard deviations were additional parameters that were estimated from the data. We used the *R-stan* package in *R* to interface and compile the *Stan* scripts that encoded the priors, model definitions, and the sampling and fitting procedures.

Comparing models

The assessment of a model's utility depends on how accurately it explains a given dataset (measured by the likelihood) as well as its ability to accurately predict new observations. A complex model with an excessive number of parameters will tend to overfit any given dataset and perform poorly when predicting new observations. On the other hand, a model that is too simple will fail to capture trends in the data, generate a low likelihood, and will also make poor predictions of new observations. The Akaike Information Criterion (Akaike, 1974, Burnham and Anderson, 2002) is commonly used to identify the model(s) within a set of alternatives that provide the best trade-off(s) between likelihood and complexity. However, the AIC penalises all model parameters equally, which may not be appropriate when they differ in their ability to influence a fit. In this study, we use the Leave-one-out information criterion (LOO-IC; Vehtari et al. (2017)) which penalizes the addition of model parameters only to the extent that they are informed by the data and so can contribute to overfitting.

Briefly, we define the log predictive density of a single observation y_i given a model with parameters θ – that is, the average value of the log-likelihood $\log\big(p(y_i|\theta)\big)$ across the joint posterior distribution of θ. We approximate this by making D draws from the posterior distribution, calculating the likelihood of y_i for each set of parameters, and averaging. This process is repeated for each data point $(y_1, ..., y_n)$ to calculate the log point-wise predictive density (lppd) for the whole dataset:

$$\text{lppd} = \sum_{i=1}^{n} \log\left(\frac{1}{D}\sum_{d=1}^{D} p(y_i|\theta^{d}_{\text{post}})\right). \tag{S10}$$

One then uses the leave-one-out (*loo*) method, a special case of cross-validation, whereby the dataset of n observations $(y_1, ..., y_n)$ is partitioned into n training datasets each of size $n-1$. Fitting the model to the training sample that excludes datapoint i gives a joint posterior $\theta^{(\text{-}i)}_{\text{post}}$. This posterior is then used to estimate the prediction accuracy of the model for the excluded observation i (the test sample), which is defined as the log of the average likelihood of the test sample across the posterior distribution. This likelihood is approximated by averaging over D draws from the posterior. This process is repeated, making each observation in the dataset $(y_1, ..., y_n)$ the test sample, and the lppd^{loo} is defined to be the sum of the log likelihoods of all these prediction accuracies:

$$\text{lppd}^{loo} = \sum_{i=1}^{n} \log\left(\frac{1}{D}\sum_{d=1}^{D} p(y_i|\theta^{(\text{-}i)d}_{\text{post}})\right) \tag{S11}$$

where the term in large parentheses characterizes the D posterior simulations fitted on $n-1$ observations when the ith observation is left out. The information criterion LOO-IC is defined as $-2 \times$ lppd (Vehtari et al., 2017). To calculate it we use the *loo-2.0* package in the *rstan* library, which estimates the lppd^{loo} using Pareto-smoothed importance sampling (PSIS) – an approximation of leave-one-out cross-validation that uses existing posterior draws from the model fits (Vehtari et al., 2015).

We then used the estimated LOO-IC values to assess the relative support for models using the analog of the Akaike weight – the probability that a given model will explain new data better than other models considered in the analysis. Following Burnham and Anderson (Burnham and Anderson, 2002), these weights are

$$w_i = \frac{\exp(-\frac{1}{2}\Delta\text{LOO-IC}_i)}{\sum_{m=1}^{M} \exp(\frac{1}{2}\Delta\text{LOO-IC}_m)}, \tag{S12}$$

where ΔLOO-IC$_i$ is the difference in LOO-IC values between model i of M candidates and the model with the lowest LOO-IC value.

E – Modelling the development of FM B cells in young mice

Empirical description of T1 precursor numbers in young mice

To capture the dynamics of T1 cells in young mice (Fig. 5A) we used the empirical function $S(t) = S_0\,(1 + t^n\,\exp(-bt))$, and fitted this to the log-transformed cell counts, using least squares to estimate S_0, n and b.

Explaining the developmental dynamics of FM B cells in young mice

To test the hypothesis of lower recruitment of T1 B cells in neonates than in adults, we allowed the rate of influx to increase with time early in life, approaching the value ψ estimated from our best-fitting model in adults aged 7 weeks and older; we assumed the form $\psi(t) = \psi(1 - \exp(-r_\psi t))$. The estimated rate r_ψ was sufficiently large that $\psi(t)$ was very close to ψ at age 7 weeks (Fig. 5E).

To test whether FM B cells in young mice are lost more rapidly than those in adult mice, we extended the time-dependent loss model, in which we had described the loss rate from age t_0=7 weeks onwards as $\delta(t) = \delta_0 e^{-r(t-t_0)}$. For $t < t_0$ we assumed $\delta(t) = \delta_0(1 - e^{r_n(t-t_0)})$ with $r_n > r$ (Fig. 5F).

We fitted both extensions of the time-dependent loss model to the counts of FM B cells in young mice separately, estimating r_ψ and r_n in the process.

Estimating the age distribution of FM B cells in young mice

To generate the predicted age distributions of cells under the two models above, we recast the models as partial differential equations (PDEs) that explicitly track cell age. In the time-dependent loss model, the population density of FM B cells of age a in mice of age t is given by the solution to

$$\frac{\partial N(a,t)}{\partial a} + \frac{\partial N(a,t)}{\partial t} = -(\delta(t) - \alpha)\,N(a,t). \tag{S13}$$

The rate of influx of cells of age zero $N(0,t)$ is the source influx $\psi S(t)$, or $\psi(t)S(t)$ for the model in which the *per capita* influx rate varies with age. The other boundary condition is the age distribution of cells at time zero, size $N(a,0)$. We assumed that the FM B cell compartment at the time of birth is sufficiently small that we could set $N(a,0) = 0$. We solved this model using the parameters estimated from fitting the extensions of time-dependent loss model (described above) to the total counts of FM B cells in young mice. We then calculated the normalised

cell age distribution of FM B cells at $t = 7$ weeks using

$$G(a,t) = \frac{N(a,t)}{\int_0^t N(a,t)da}.$$

See Rane et al. (2018) for full details of the solution of this class of model.

Modelling the distribution of GFP expression in FM B cells

We assumed that the GFP intensity within FM B cells declines exponentially with their age, since there is no residual Rag activity from the T1 stage onwards (Yu et al., 1999) and the estimated slow rate of self-renewal of FM B cells means that dilution of GFP through division is minimal. Newly matured FM B cells of age $a = 0$ therefore have GFP intensity f_{max}, which then declines as $f(a) = f_{\text{max}}e^{-\gamma a}$, and f_{max} and γ are parameters to be estimated. There is therefore a 1-to-1 correspondence between GFP expression f and cell age a, and so we recast the FM B cell population density $N(a,t)$ (the solution to eqn. S13) as

$$\begin{aligned} N(f,t) &= N(a,t)\left|\frac{da}{df}\right| \\ &= N\left(\frac{\ln f_{\text{max}} - \ln f}{\gamma}, t\right)\frac{1}{\gamma f} \end{aligned} \tag{S14}$$

where the Jacobian da/df preserves local cell densities under coordinate transformations. The mean fluorescence intensity (MFI) of GFP in FM B cells in a mouse of age t is then

$$\text{MFI}(t) = \frac{\int_{f_{\text{min}}}^{f_{\text{max}}} f\,N(f,t)\,df}{\int_{f_{\text{min}}}^{f_{\text{max}}} N(f,t)\,df}, \tag{S15}$$

where f_{min} is a lower cutoff in GFP expression needed to avoid divergence in the integral (GFP never decays to zero) and was set to 0.1, well below all values observed experimentally. For each model, we estimated f_{max} and γ by fitting eqn. S15 to the observed timecourse of the MFI of GFP in FM B cells. Using the solution $N(f,t)$ for each model we then predicted the timecourse of the MFI within GFP^{pos} FM B cells only, using a gating threshold of $f_{\text{min}} = 1000$.

F – Using information from Ki67-reporter mice to aid discrimination between models of GC B cell dynamics

The Ki67-Cre-ER^{T2}-YFP system allows us to track cohorts of cells that underwent cell division during tamoxifen treatment. We measured the frequencies of YFP-expressing cells at day 4 and day 62 post-tamoxifen and, for each model (as described below), used the decline in YFP expression over this time period to constrain the rates of loss and/or division. YFP expression is preserved upon cell division but is diluted by loss or onward differentiation. To illustrate, for the simple homogeneous model, YFP expression will decline at the net rate of loss of the population λ, which is $\delta - \alpha$. We can therefore relate λ to the fold loss of YFP expression over a time τ:

$$Y(t) = Y_0\,e^{-\lambda\tau} \implies \lambda = -\log(\Delta Y)/\tau. \tag{S16}$$

Priors on ΔY. We took the strategy of making ΔY a parameter in the model, using its observed values to generate its prior; and sampling from this prior then allowed us to estimate or constrain other parameters. For splenic GC B cells, the mean YFP-labelled fraction dropped from 0.35 to 0.04 over 8 weeks, yielding $\Delta Y \sim 0.12$. This, together with the scatter in ΔY observed in YFP reporter mice (4 mice at the 2 week timepoint and 5 mice at the 8 week timepoint, unpaired data; Fig. 6D in the text), suggested $\Delta Y \sim \mathcal{N}(0.12, 0.05)$. Lymph node GC B cells exhibited

$\Delta Y \simeq 0.42$. When assuming T1 or T2 as their precursors, which turn over rapidly and are therefore not expected to provide a persistent source of YFP-labelled cells after withdrawal of tamoxifen, we therefore assumed $\Delta Y \sim \mathcal{N}(0.42, 0.05)$. When assuming FM B cells to be precursors, which turn over more slowly, we considered the possibility that FM B cells might act as a reservoir that feeds new YFP$^+$ cells into LNGC for some time after withdrawal of tamoxifen. In the case the drop in YFP expression yields only a lower bound on λ. Accordingly, we assumed that ΔY was skew-normally distributed with a bias towards values less than the mean of 0.42, and a standard deviation of 0.1 ($\Delta Y \sim$ SkewNormal$(0.42, 0.1, -5)$). Specifically, if $\mu \in \mathbb{R}, \sigma \in \mathbb{R}^+$, and $k \in \mathbb{R}$, then for y $\in \mathbb{R}$,

$$\text{SkewNormal}(y \mid \mu, \sigma, k) = \frac{1}{\sigma\sqrt{2\pi}}\, e^{-\frac{(y-\mu)^2}{2\sigma^2}} \left(1 + \text{erf}\left(k(\frac{y-\mu}{\sigma\sqrt{2}})\right)\right),$$

where 'erf' is the Gaussian error function. For each model we used the YFP information in the following ways:

Simple homogeneous model (with or without time-dependent influx): Using the above priors on ΔY and the division rate α, we then estimated the rate of loss (δ) using equation S16;

$$\delta = \frac{-\log(\Delta Y)}{\tau} + \alpha.$$

Time-dependent division or loss: With time dependent division, we assumed priors for ΔY and δ and calculated the rate of division at t_0, which we denote α_0. With time dependent loss loss, we assumed priors for ΔY and α and calculated the rate of loss at t_0 (δ_0):

$$\begin{aligned} \alpha_0 &= \frac{\log(\Delta Y) + \delta\tau}{\tau\, e^{\tau\, r}} \\ \delta_0 &= \frac{-\log(\Delta Y) + \alpha\tau}{\tau\, e^{\tau\, r}}. \end{aligned} \tag{S17}$$

Kinetic heterogeneity: This model (eqns. S3) predicts a biphasic loss of YFP, reflecting the net loss rates of the transient (λ_f) and persistent (λ_s) subsets and which were present at unknown frequencies q and $1-q$, respectively. We then assigned priors ΔY, λ_f and λ_s, to give q:

$$\begin{aligned} Y(\tau) &= Y_0^f\, e^{-\lambda_f\,\tau} + Y_0^s\, e^{-\lambda_s\,\tau} \\ \Delta Y = Y(\tau)/Y(0) &= q\, e^{-\lambda_f\,\tau} + (1-q)\, e^{-\lambda_s\,\tau} \\ \implies q &= \frac{\Delta Y - e^{-\lambda_s\,\tau}}{e^{-\lambda_f\,\tau} - e^{-\lambda_s\,\tau}}. \end{aligned} \tag{S18}$$

Here Y_0^f and Y_0^s are the fractions of YFP-labelled cells in the transient and persistent subsets, respectively, at time $\tau = 0$. Assuming $\lambda_f > \lambda_s$, the constraint $0 < q < 1$ in turn constrains the priors on λ_f and λ_s;

$$\lambda_s < \frac{-\log(\Delta Y)}{\tau} < \lambda_f.$$

Placing priors on the division rates α_f and α_s, we could then calculate the rates of loss of transient (δ_f) and persistent subsets (δ_s) using

$$\begin{aligned} \delta_f &= \lambda_f + \alpha_f \\ \delta_s &= \lambda_s + \alpha_s. \end{aligned}$$

Incumbent: We derived a similar relationship between $\lambda_{\text{displaceable}}$ and $\lambda_{\text{incumbent}}$ to that in eqn. S18,

$$\lambda_{\text{incumbent}} < \frac{-\log(\Delta Y)}{\tau} < \lambda_{\text{displaceable}}.$$

Using priors on ΔY, $\lambda_{\text{displaceable}}$, $\lambda_{\text{incumbent}}$, α_{inc} and α, we calculated δ and δ_{inc}:

$$\delta = \lambda_{\text{displaceable}} + \alpha$$
$$\delta_{\text{inc}} = \lambda_{\text{incumbent}} + \alpha_{\text{inc}}.$$
