# Supplementary material for: Fate Mapping Quantifies the Dynamics of B Cell Development and Activation throughout Life
Source: Cell Rep. Author manuscript; Available in PMC 2021 Nov 26. (PMC8622872; doi:10.1016/j.celrep.2020.108376)
Supplement: 3 [file NIHMS1647629-supplement-3.pdf]

Cell Reports

Article

Fate Mapping Quantifies the Dynamics of B Cell Development and Activation throughout Life

Graphical Abstract

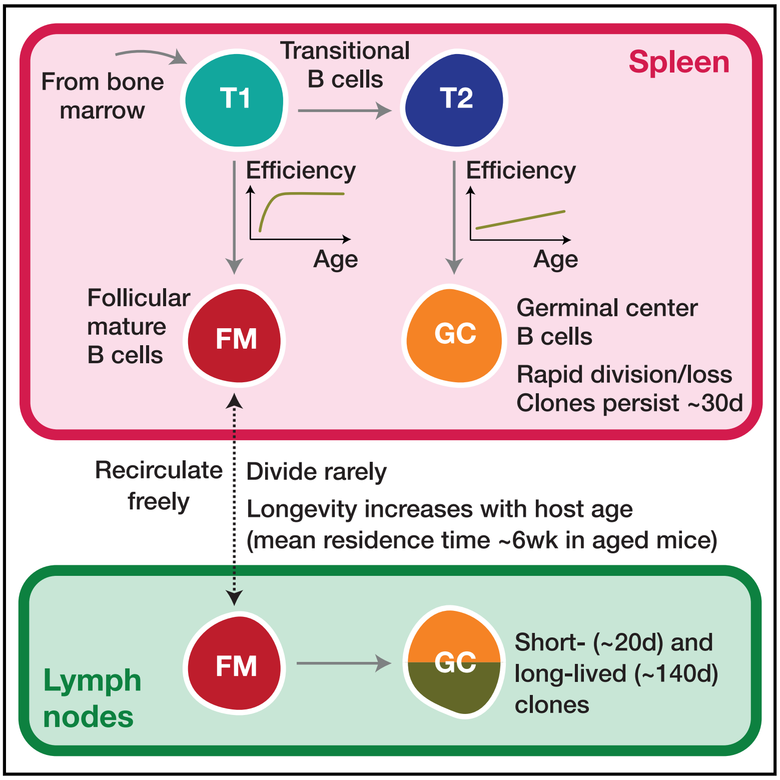

Authors

Melissa Verheijen, Sanket Rane, Claire Pearson, Andrew J. Yates, Benedict Seddon

Correspondence

andrew.yates@columbia.edu (A.J.Y.), benedict.seddon@ucl.ac.uk (B.S.)

In Brief

Verheijen and Rane et al. combine fate mapping and mathematical models to quantify the development and dynamics of follicular mature B cells and germinal center B cells in spleen and lymph nodes, and show how these processes vary across the mouse lifespan.

Highlights

- Follicular mature B cells are a homogeneous population that self-renews infrequently
- Host environment drives cell-extrinsic, age-related changes in B cell homeostasis
- Clones in naturally occurring germinal centers (GC) persist for weeks with rapid turnover
- GC B cells in spleen and lymph nodes have different precursors and distinct dynamics

Verheijen et al., 2020, Cell Reports *33*, 108376
November 17, 2020 © 2020 The Authors.
https://doi.org/10.1016/j.celrep.2020.108376

CellPress

Cell Reports

CellPress
OPEN ACCESS

Article

Fate Mapping Quantifies the Dynamics of B Cell Development and Activation throughout Life

Melissa Verheijen,[1,4] Sanket Rane,[3,4] Claire Pearson,[2] Andrew J. Yates,[3,*] and Benedict Seddon[1,5,*]
[1]Institute of Immunity and Transplantation, Division of Infection and Immunity, UCL, Royal Free Hospital, Rowland Hill Street, London NW3 2PF, UK
[2]Kennedy Institute of Rheumatology, University of Oxford, Oxford, UK
[3]Department of Pathology and Cell Biology, Columbia University Medical Center, 701 West 168th Street, New York, NY 10032, USA
[4]These authors contributed equally
[5]Lead Contact
*Correspondence: andrew.yates@columbia.edu (A.J.Y.), benedict.seddon@ucl.ac.uk (B.S.)
https://doi.org/10.1016/j.celrep.2020.108376

SUMMARY

Follicular mature (FM) and germinal center (GC) B cells underpin humoral immunity, but the dynamics of their generation and maintenance are not clearly defined. Here, we exploited a fate-mapping system in mice that tracks B cells as they develop into peripheral subsets, together with a cell division fate reporter mouse and mathematical models. We find that FM cells are kinetically homogeneous, recirculate freely, are continually replenished from transitional populations, and self-renew rarely. In contrast, GC B cell lineages persist for weeks with rapid turnover and site-specific dynamics. Those in the spleen derive from transitional cells and are kinetically homogeneous, while those in lymph nodes derive from FM B cells and comprise both transient and persistent clones. These differences likely derive from the nature of antigen exposure at the different sites. Our integrative approach also reveals how the host environment drives cell-extrinsic, age-related changes in B cell homeostasis.

INTRODUCTION

The ability to mount effective humoral immune responses throughout life is critical for normal antibody-mediated protection and healthy aging (Gibson et al., 2009; Frasca et al., 2011). B cells are generated in the bone marrow (BM) and enter the spleen where they complete development as transitional cells, characterized by the induction of CD23 and immunoglobulin D (IgD), together with downregulation of IgM and AA4.1 (Allman et al., 2001; Loder et al., 1999). These markers identify three stages of transitional cell maturation. During the T1 stage, IgM^{hi} CD23^{low} B cells with autoreactive B cell receptors (BCRs) undergo negative selection (Petro et al., 2002; Su and Rawlings, 2002). During the T2 stage, CD23^{hi}IgD^{hi} cells commit to either a follicular B cell fate, progressing through the IgM^{low} T3 stage or are diverted to develop into marginal zone B cells, losing expression of CD23, upregulating IgM, and expressing CD21 (Petro et al., 2002; Su and Rawlings, 2002; Lam et al., 1997; Pillai and Cariappa, 2009; Torres et al., 1996). Follicular mature (FM) B cells recirculate between lymph nodes (LNs) and spleen, where cognate encounter with antigen triggers activation and the development of germinal center (GC) reactions. In deliberately challenged mice, antigen-specific GC B cells divide extensively and undergo affinity maturation (Mesin et al., 2016; Basso and Dalla-Favera, 2015; De Silva and Klein, 2015). However, GC B cells are present throughout a mouse's lifetime even in the absence of deliberate immunological challenge. The origin and dynamics of these constitutive GC reactions are not well characterized.

While the establishment of peripheral B cell subsets relies upon *de novo* generation in the BM, it is unclear to what degrees the processes of influx of new cells, proliferative renewal, and cell loss (turnover) combine to maintain B cell subsets at or close to equilibrium, and how these processes may change throughout life. Much of our insights into these dynamics derive from DNA labeling experiments using bromodeoxyuridine (BrdU). Numbers of immature B cells in the spleen decline with age, and it has been inferred from BrdU labeling that this decline derives from a loss of efficiency of pre-transitional B cell development, rather than any decrease in the rate of production of B cell progenitors in the BM (Kline et al., 1999; Shahaf et al., 2006). In adult mice, it has been estimated that approximately 4×10^5 cells enter the mature naive (FM) B cell pool daily (Srivastava et al., 2005), which is approximately 1% of the total pool size. This low rate implies that if FM B cells are maintained at roughly constant numbers, the average, net rate of turnover (the balance of loss and any self-renewal) must also be low, and indeed it has been observed that only around 50% of FM B cells are replaced over a period of 12 weeks in adults (Förster and Rajewsky, 1990; Fulcher and Basten, 1997). BrdU labeling

This is an open access article under the CC BY license (http://creativecommons.org/licenses/by/4.0/).

studies have also indicated that the average lifespan of mature B cells increases with age, an effect that acts to compensate for the decrease in their precursor numbers (Kline et al., 1999; Fulcher and Basten, 1997).

In regard to GC reactions, much attention has focused on the dynamics associated with affinity maturation, the factors influencing transitions between light and dark zones, and the associated processes of proliferation and differentiation (see, for example, Victora and Mesin [2014] and Mesin et al. [2016]). However, less is known regarding the population dynamics and rules of replacement of GC B cells over extended timescales. In particular, it is unclear how division and death combine to define the longevity of GC reactions, and whether their population dynamics are sensitive to host age.

Quantifying these balancing acts is important, not only for understanding how to treat dysregulation of B cell homeostasis, but also for understanding how B cell repertoires evolve over time. In this setting, one must be careful to distinguish between the lifespans of individual cells, and the persistence of populations that self-renew. Collectively the descendants of a naive B cell (which comprise a B cell lineage, that we loosely refer to as a ‘‘clone’’) may persist much longer than any individual cell. This clonal lifespan is the pertinent quantity when measuring the persistence of antigen-specific B cell populations. Blocking B cell development by interfering with interleukin-7 (IL-7) signaling or with inducible deletion of Rag2 has indicated that mature B cell populations can persist without influx for weeks to months (Grabstein et al., 1993; Hao and Rajewsky, 2001), timescales that reflect average clonal lifespans.

Insights from BrdU labeling studies can be limited due to its toxicity in the longer term and potential spatial heterogeneity in the efficiency of its uptake. Also, the use of irradiated chimeras to monitor the dynamics of repopulation and maintenance is complicated by the lymphopenic environment, which induces transitional cells to undergo homeostatic proliferation (Meyer-Bahlburg et al., 2008). Careful quantification of cell population dynamics from BrdU labeling experiments also requires the use of mathematical models, and estimates of key quantities such as division and turnover (loss) rates can be sensitive to the assumptions encoded in these models (De Boer et al., 2003; De Boer and Perelson, 2013). For instance, labeling curves are often multi-phasic, indicative of heterogeneity in rates of proliferation, but fully resolving and quantifying this heterogeneity can be difficult. It can also be problematic to distinguish labeling derived from proliferation within a cell subset and from the influx of labeled cells from a precursor population, and to distinguish between potential precursor populations. To address all of these issues, here we employed the method of temporal fate mapping (Hogan et al., 2015) to characterize the population dynamics and the rates and extents of tonic reconstitution of FM and naturally occurring GC B cell compartments in healthy mice. We studied the kinetics by which new B cells percolate into peripheral subsets, and paired this information with measures of proliferation (Ki67 expression), accounting for its possible persistence across stages of development. We then confronted these data with an array of candidate mathematical models, to identify the most concise and robust descriptions of the ontogeny and dynamics of FM and GC B cells over almost the full extent of the mouse lifespan.

RESULTS

Busulfan Treatment Permits Reconstitution of the BM HSC Niche without Perturbing Peripheral Mature B Cell Compartments

To study the dynamics of FM and GC B cells, we used a previously published method of tracking lymphocyte development in healthy mice (Hogan et al., 2015). Briefly, treatment with optimized doses of the transplant conditioning drug busulfan ablates the host hematopoietic stem cell (HSC) compartment but has no impact on mature peripheral hematopoietic lineages. We then transfer congenically labeled HSC progenitors from donor BM, which rapidly reconstitute the depleted host HSC niche. This procedure typically achieves 60%–95% replacement of HSC, which remains stable for the lifespan of the mouse. We then follow the replacement of mature peripheral hematopoietic compartments by the progeny of donor HSC for up to 18 months post-BMT. The kinetics of the infiltration of donor cells into the intact peripheral lymphocyte subsets are rich in information regarding differentiation pathways, the fluxes between subsets and the net rates of loss within each, and the rules of replacement (Hogan et al., 2015; Gossel et al., 2017; Hogan et al., 2019).

Specifically, we generated chimeras by conditioning CD45.1 C57Bl6/J hosts with busulfan and reconstituting HSC with T and B cell depleted BM from CD45.2 C57Bl6/J donors (STAR Methods). To help us evaluate any influence of host age on B cell maintenance, we generated chimeras using hosts of varying ages, partioned into three groups: 8 weeks, 8–12 weeks, and ages 12 weeks or older. Previously, we showed that our busulfan conditioning regime has no detectable impact upon the long-term survival, proliferation, or maintenance of peripheral T cell compartments or their progenitors (Gossel et al., 2017; Hogan et al., 2015). To confirm this was also true for B cells, we compared peripheral B cell subsets (Figure 1A) in busulfan-treated mice with age-matched WT controls at different times following BM reconstitution. We saw no significant differences in total numbers of transitional, FM, or GC B cells (Figure 1B) or in their levels of expression of Ki67, a marker of recent division (Figure 1C), in either spleen or LNs in the weeks and months following BMT.

To account for variation between busulfan-treated mice in the extent of HSC replacement after BMT, we used the mouse-specific values of the chimerism among equilibrated progenitor populations to normalize the levels of donor cell infiltration into downstream peripheral B cell subsets. With this approach, a normalized chimerism of 1 indicates that a subset has attained the chimerism of its ancestral population, meaning that it has turned over completely. In our previous studies of T cell homeostasis in busulfan chimeras, peripheral donor chimerism was normalized to the equilibrated chimerism in thymic progenitors (Hogan et al., 2015). A similar approach normalizing against donor chimerism among BM B cell progenitors was not possible, because we observed substantial variation in donor engraftment between different bones in the same mouse (Figure S1A). No single site was therefore representative of the entire BM

CellPress
OPEN ACCESS

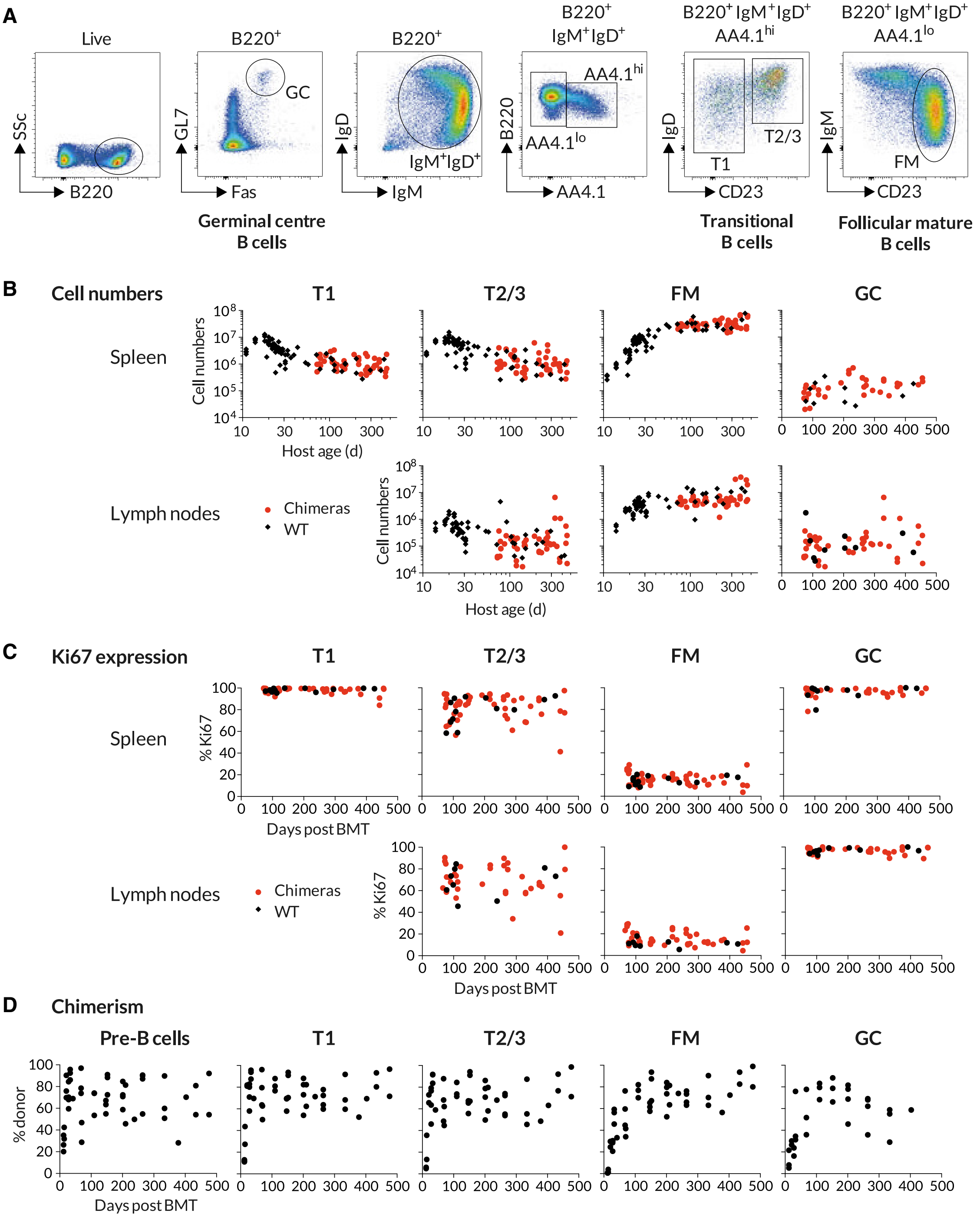

Figure 1. Busulfan Chimeric Mice Exhibit Normal Peripheral B Cell Compartments

Busulfan chimeras were generated as described in STAR Methods (n = 47) and compared with WT controls (n = 74). Data are pooled from multiple experiments.

(A) Gating strategy to identify transitional, follicular mature, and germinal center B cells.

(B) Comparing the sizes of B cell subsets in WT control mice and busulfan chimeras.

(C) Comparing proliferative activity in WT and busulfan chimeric mice, using Ki67 expression.

(D) Host-derived B cells are gradually replaced by donor-derived cells over time. Scatter derives largely from variation in levels of stable bone marrow chimerism achieved in treated mice.

See also Figure S1.

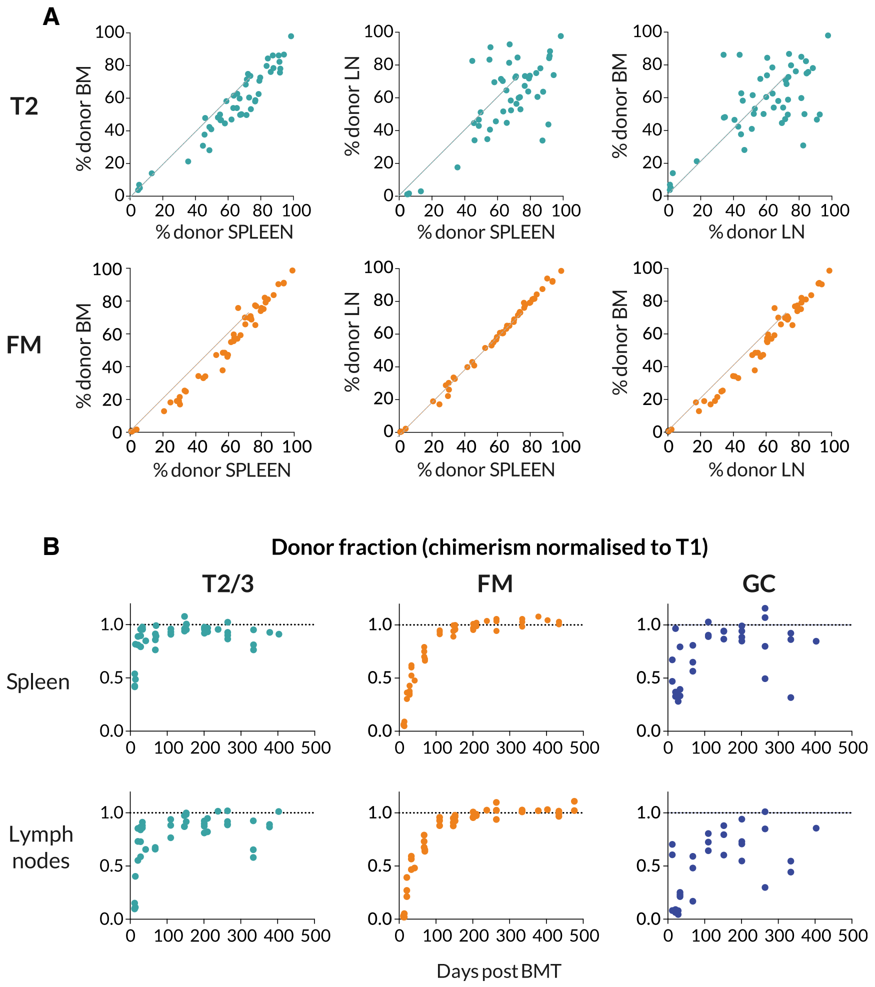

Figure 2. Spatial Distribution and Dynamics of Chimerism in B Cell Subsets

(A) Comparing chimerism in the T2 and FM B cell subsets across bone marrow (BM), spleen, and pooled lymph nodes (LNs), at multiple time points.

(B) The kinetics of accumulation of donor-derived cells in B cell subsets. The donor fraction in each subset is normalized to that in the upstream T1 subset, to remove the effect of variation in the level of stable BM chimerism achieved across animals.

compartment. However, all developing B cells migrate from BM to the spleen to continue development as AA4.1$^+$IgM^{hi}CD23^{lo} (transitional) cells. Since this obligate stage integrates input from all BM sites, we used the chimerism among these cells (referred to as T1) as a proxy for the chimerism across total BM progenitors. In the few weeks following BMT, we observed a smooth transition from exclusively host-derived to donor-enriched cells within BM pre-B cells and the spleen-resident transitional populations (Figure 1D), indicating that all turn over rapidly. There followed a slower emergence of chimerism among FM or GC B cells. Importantly, we then saw no trend in donor chimerism among pre-B or T1 cells across animals over time (Figure 1D), suggesting that BM chimerism, once established, was stable, consistent with our previous studies (Hogan et al., 2015).

Temporal Fate Mapping Reveals Extensive Replacement of Mature B Cell Compartments by HSC Progeny following BMT

We observed a high degree of correlation in donor chimerism among CD23^{hi} transitional 2/3 (T2/3) and FM B cells in LN, spleen, and BM in each mouse (Figure 2A), confirming that these mature B cell populations are freely recirculating between the different lymphoid sites. As anticipated, the T2/3 compartment underwent complete replacement within a few weeks following BMT (Figure 2B).

The replacement kinetics of GC B cells in spleen and LNs were somewhat more noisy (Figure 2B), but there appeared to be more rapid replacement within splenic GC than in LNs. Since GC B cells are non-recirculating, we assumed that these two locations contained independent populations of activated B cells. Indeed, analyzing individual LN separately revealed considerable variation in chimerism among GC B cells within a single host (Figure S1B), suggestive of a degree of stochasticity with which donor or host cells are recruited to GC reactions. Nevertheless, for subsequent analyses we pooled LNs in order to measure overall levels of donor infiltration, but treated LN and spleen separately. We reasoned that the origins of stimuli driving GC formation in LN and spleen, deriving from tissue drainage and blood, respectively, might result in qualitatively distinct responses.

Quantifying Cell Production: Ki67 Expression Reflects Self-renewal but Is Also Inherited across Stages of B Cell Development

To examine the role of proliferation in B cell development, we also measured levels of Ki67, a nuclear protein that serves as a marker of recent cell division. In T cells, Ki67 expression is induced at the G1 stage of the cell cycle and persists for more than 3 days after mitosis (Gossel et al., 2017; Hogan et al., 2013). Similar decay kinetics have been described in human mammary epithelial cell lines *in vitro* (Miller et al., 2018).

Analysis of Ki67 expression revealed variation across stages of B cell development in BM, transitional stages, and mature B cells (Figure 3A). Pre-B cells and GC B cells both undergo extensive proliferation and exhibited a high, unimodal distribution of Ki67 expression. It was also readily detectable among transitional B cells but was expressed by only a small subset of FM B cells (Figure 3A). It has been shown that transitional populations in the spleen do not divide as they mature and that,

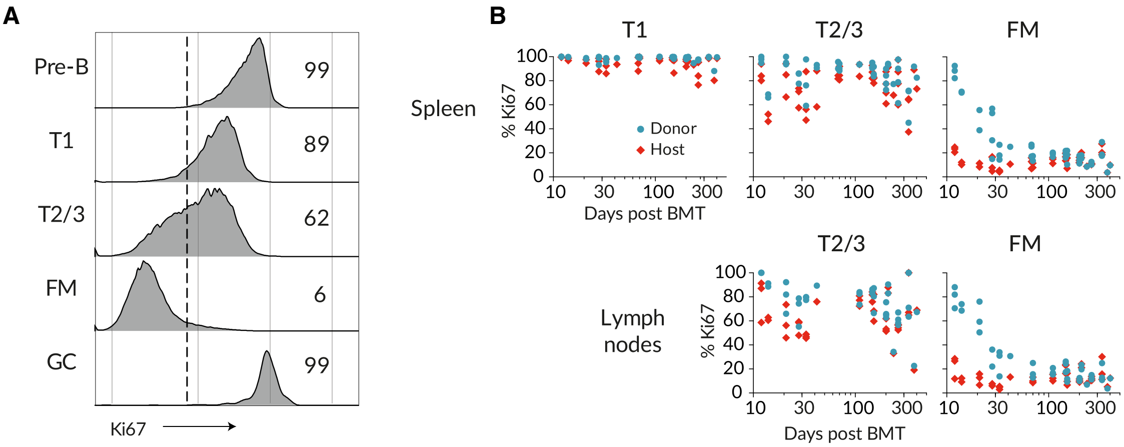

Figure 3. Levels of Proliferation Vary throughout B Cell Development

(A) Ki67 expression in different B cell subsets; pre-B cells in bone marrow, transitional T1/2/3 cells in spleen, and FM and GC B cells in spleen.

(B) Ki67 frequencies among donor- and host-derived B cell subsets with time post BMT.

following BrdU administration, labeled T2 cells are detected in the spleen within 2 days (Srivastava et al., 2005), a maturation time that is shorter than the lifetime of Ki67. Therefore, Ki67 seen in transitional populations is likely residual expression from division events among precursors in the BM. Consistent with this inference, Ki67 abundance in transitional populations remains broadly unimodal in distribution, but its median level falls as they mature into FM B cells (Figure 3A). Given the short transit time through the transitional stages, it is possible that the low level of Ki67 expression in FM B cells (Figure 1B and Figure 3A) could also derive at least in part from recently divided BM precursors, as well as from self-renewal. Observing the influx of new donor cells revealed evidence of inheritance of Ki67 in the FM B cell pool. Soon after BMT, donor FM B cells exhibited elevated levels of Ki67 relative to host cells, but these declined to host levels after approximately 100 days (Figure 3B). We infer that this dynamic derives naturally from the difference in the mean ages of donor and host B cells, which is more pronounced soon after BMT when all donor-derived FM cells have recently entered the compartment; the dynamic does not derive from any intrinsic differences in the behavior of host and donor cells. Therefore, in the modeling analyses described below, we assumed that Ki67 expression within each B cell subset could derive from division and/or the influx of Ki67^{hi} progenitors.

FM B Cells Are a Homogeneous, Slowly Dividing Population Whose Residence Time Increases with Age

Given the time-varying fluxes of donor cells through multiple stages of development, extracting the maximum information from these time courses requires mathematical modeling. We have previously used this approach using busulfan chimeric mice to quantify the developmental and homeostatic dynamics of naive CD4 and CD8 T cells (Hogan et al., 2015) and memory CD4 T cells (Gossel et al., 2017; Hogan et al., 2019).

We began by studying FM B cells. We assumed that they recirculate freely between LNs and spleen, given the close similarity in chimerism in the two compartments (Figure 2A) and indeed across all lymphoid organs (Figure S1B). Therefore we pooled the numbers of FM B cells recovered from spleen and LNs and assumed they follow the same dynamics in each. We attempted to describe these dynamics in mice aged between 70 and 600 days with a variety of mathematical models (Figure 4A). In each, we assumed newly differentiated FM B cells are generated at a rate proportional to the size of their precursor population, which was assumed to be T1, T2, or T1 and T2 combined. Describing the time courses of these "source" populations with empirical functions (Figure S2; Methods S1, part A), we then aimed to identify the combination of model and precursor population that best described FM B cell dynamics. The simplest model (Figure 4A, top) assumed that FM B cells, whether host or donor, are generated from their precursors at the same constant *per capita* rate and form a homogeneous population that undergoes turnover (loss) and self-renews through division, both at constant *per capita* rates. This model predicts a smooth, continuous approach to stable chimerism of FM B cells with eventual complete (and repeated) replacement. However, the precise shape of this curve is rich in information regarding the processes of influx and loss. To test for any more complex homeostatic dynamics, we considered four extensions to this basic model. In the first, the rates of turnover or division might vary with host age (the "time-dependent turnover" or "time-dependent division" models). In the second, the FM B cells are assumed to be homogeneous with constant rates of turnover and division but are fed from transitional B cells at a *per capita* rate that changes with age ("time-dependent recruitment"). In the third extension, FM B cells comprise two independent subpopulations turning over at different rates ("kinetic heterogeneity"). In this scenario, the donor chimerism will initially increase rapidly as the subpopulation with faster turnover is replaced, followed by a more gradual approach to stable chimerism as the more persistent subpopulation, with slower turnover, is replaced. In the fourth extension (the "incumbent" model), we allowed for the possibility that a population of host-derived cells established early in life remains stable in numbers and is not replaced by cells recruited

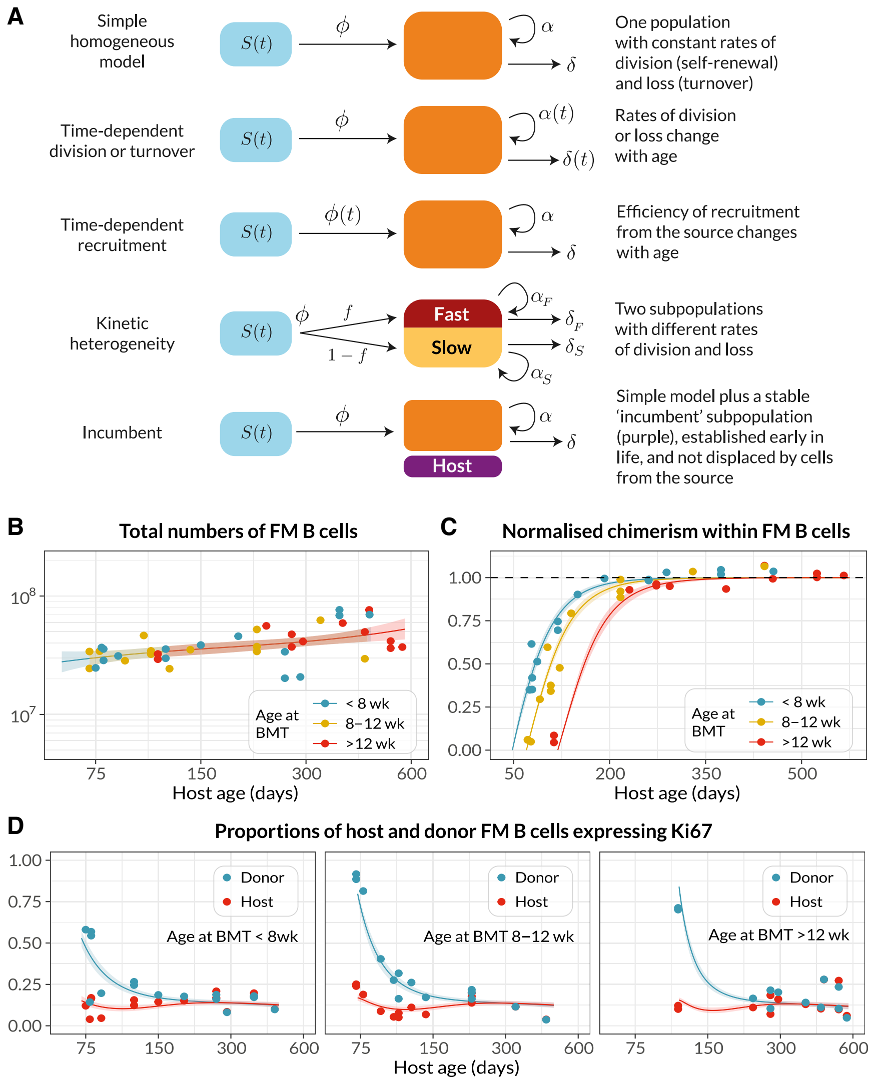

Figure 4. Modeling the Population Dynamics of FM B Cells in Busulfan Chimeric Mice

(A) Schematics of the candidate mathematical models of FM B cells (orange) fed by a precursor population (blue).

(B–D) Observed (points) and best-fit values (lines) of (B) total FM B cell numbers, from pooled spleen and lymph nodes; (C) their chimerism, normalized to that in T1; and (D) the proportions of host and donor FM B cells expressing Ki67. Lines show the predictions from the best-fitting (time-dependent turnover) model, in which the mean residence time of FM B cells increases with mouse age. Colors in (B) and (C) denote groups of mice who underwent BMT at different ages: <8 weeks (n = 15), 8–12 weeks (n = 14), >12 weeks (n = 12). Shaded regions are prediction intervals, generated by drawing samples from the posterior distribution of parameter estimates and plotting the 4.5 and 95.5 percentiles of the resulting model predictions. Fitted values were specific to each mouse, with its particular age at BMT; model predictions shown here were generated using the mean age at BMT within each group.

See also Figures S2 and S3.

later in life. Such a model allows for less-than-complete turnover, or a normalized chimerism stabilizing at a value less than 1. See Methods S1, part B for details of the mathematical formulation of the models.

The kinetic of replacement of existing cells with immigrant cells is determined primarily by the average net rate of loss—the balance of cell death, any onward differentiation, and proliferative renewal (Methods S1, part C). We refer to the inverse of the net loss rate as the clonal lifespan; it measures the persistence of a population of B cells that is subject to both loss and any degree of self-renewal. The clonal lifespan may be much longer than the expected time any one cell spends within that population before it dies or differentiates, which we refer to as the residence time.

To estimate parameters and compare the support for the models, each was fitted simultaneously to the time courses of FM B cell numbers, the chimerism within FM B cells normalized to that in T1 (the earliest common precursor to all populations considered), and the proportions of host and donor FM B cells expressing Ki67 (Figures 4A–4C). Including the Ki67 expression in donor and host populations allowed us to resolve the net loss rate into rates of death/differentiation (yielding the residence time) and proliferation (yielding the interdivision time). Our approach to fitting is described in detail in Methods S1, part D.

We could immediately reject the incumbent model, because the chimerism of the FM B cell compartment reached that of T1 cells (Figure 4B), indicating no evidence for a persistent host-derived FM B cell population. We found the strongest relative support (64%, Table S1) for the model in which the rate of turnover of FM B cells declined with host age but their rate of division remained constant, and with T1 cells as their direct precursor. The fits of this model to the data are shown as solid lines in Figures 4A–4C, and parameter estimates are in Table 1. Fits using the alternative models are shown in Figure S3, and parameter estimates for all models are provided in Data S1.

We estimate a steady daily influx of around 0.9 × 10^6 new FM B cells per day from T1 precursors into the spleen and LN of mice aged between 75 and 500 days, deriving from the assumption of a constant *per capita* rate of recruitment and the relatively stable numbers of T1 precursors in this age range (Figure 1A). This flux is almost double an estimate of the number of new FM B cells entering the spleen daily in 56-day-old mice (Srivastava et al., 2005). We infer that FM B cells have a mean residence time of roughly 5 weeks in 75-day-old mice, and that this increases slowly over time (6 weeks at age 300 days, and almost 9 weeks

CellPress
OPEN ACCESS

Table 1. Parameters Governing Homeostasis of Circulating Follicular Mature B Cells and Germinal Center B Cells in the Spleen and Lymph Nodes

Population	Parameter	Estimate and 95% CI			
FM B cells	Daily influx from T1 (as % of subset) at age 75 days	2.9	(2.5, 3.4)		
	at age 300 days	2.1	(1.8, 2.4)		
	Daily influx from T1 at age 75 days (cells/day $\times 10^{-6}$)	0.87	(0.74, 1.0)		
	at age 300 days	0.85	(0.73, 0.99)		
	Mean residence time at age 75 days (d)	35	(29, 42)		
	at age 300 days (d)	42	(35, 50)		
	Time taken for mean residence time to double (months)	27	(15, 115)		
	Mean clonal lifespan at age 75 days (d)	41	(33, 50)		
	at age 300 days (d)	51	(43, 61)		
	Mean inter-division time (d)	400	(110, 1200)		
	Ki67^{hi} → Ki67^{lo} transit time (d)	5.8	(4.4, 7.2)		
GC B cells	Daily influx from T2 (as % of subset) at age 75 days	5.0	(3.3, 7.1)		
(Spleen)	at age 300 days	3.8	(2.4, 5.7)		
	Mean residence time (d)	0.55	(0.41, 0.73)		
	Mean clonal lifespan (d)	29	(23, 35)		
	Mean inter-division time (d)	0.56	(0.41, 0.75)		
	Time taken for *per capita* rate of influx to double (d)	230	(120, 680)		
	Ki67^{hi} → Ki67^{lo} transit time (d)	5.5	(4.1, 7.1)		
		Transient Subset		Persistent Subset	
GC B cells	Daily influx from FM B cells (% of subset) at age 75 days	0.63	(0.08, 2.1)	2.1	(1.2, 3.4)
(Lymph nodes)	at age 300 days	0.36	(0.03, 1.6)	1.8	(1.0, 2.7)
	Mean residence time (d)	0.51	(0.35, 0.69)	0.73	(0.42, 0.74)
	Mean clonal lifespan (d)	21	(1, 58)	140	(50, 550)
	Mean inter-division time (d)	0.58	(0.43, 0.75)	0.73	(0.53, 1.4)
	Proportion of total GC B cells at age 75 days	0.23	(0.06, 0.39)	0.77	(0.61, 0.94)
	at age 300 days	0.16	(0.03, 0.37)	0.83	(0.63, 0.97)
	Ki67^{hi} → Ki67^{lo} transit time (d)	5.9	(4.6, 7.3)	5.9	(4.6, 7.3)

95% credible intervals were estimated by taking the 2.5 and 97.5 percentiles of the posterior probability distributions of the parameter values. We inferred that the expected residence time of FM B cells (that is, the mean time until their loss or onward differentiation) increases with host age. For splenic GC B cells, the rate of influx of new cells into the compartment from T2 precursors increases with host age. We infer that lymph node GC B cells derive from FM B cells and comprise at least two subpopulations with different rates of division and loss. Both populations are assumed to share the same Ki67 lifetime.

at age 2 years). These estimates are in line with those from older studies of BrdU labeling among mature B220^{hi} HSA^{low} B cells, which are predominantly FM cells (Förster and Rajewsky, 1990; Fulcher and Basten, 1997).

While in adult mice approximately 10% of FM B cells express Ki67 (Figures 1B and 4D), we infer that this level of expression derives almost entirely from newly generated FM cells who inherit it from their pre-transitional, highly proliferative BM precursors. As described above, this conclusion derives largely from the observation that donor-derived FM B cells, which soon after BMT are highly enriched for newly generated cells, transiently exhibit significantly higher levels of Ki67 than the more established host cells (Figure 4D). We infer that FM B cells themselves divide rarely—roughly once a year, though this estimate comes with some uncertainty. Because this self-renewal is slow, the average clonal lifetime is only slightly longer than the mean residence time of individual cells themselves. Therefore, the naive FM B cell compartment in adult mice relies almost entirely on the influx of new cells—and is therefore constantly supplied with new receptor specificities—for its maintenance throughout life.

Developmental Dynamics of FM B Cells Differ in Young and Adult Mice

Next, we studied the accumulation of FM B cells early in life to understand how the dynamics of their establishment in lymphoid organs compares to their dynamics in adult mice. Their T1 precursors dramatically increased in number up to age 20 days, declined continuously for a further 20–30 days, and were maintained stably thereafter (Figure 5A). Correspondingly, FM B cell numbers increased rapidly up to age 30 days, followed by the much slower but persistent increase that we modeled in adults (Figure 5B). We wanted to explore whether the processes of generation and maintenance of FM B cells from T1 precursors that

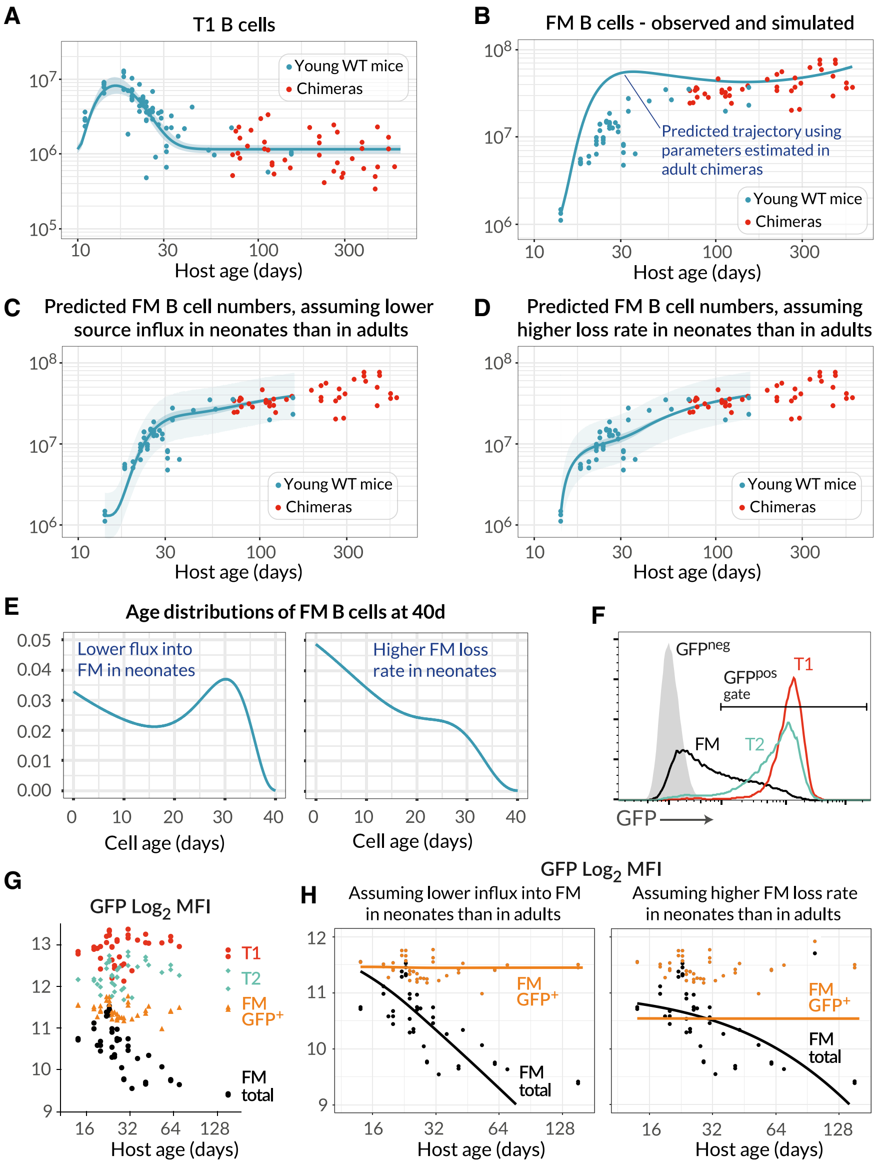

Figure 5. Establishment of the FM B Cell Compartment in Young Mice Is Characterized by Progressively Increasing Recruitment from T1 Precursors with Age

(A) Numbers of splenic T1 B cells recovered from both young WT control mice (n = 45, blue) and adult busulfan chimeras (n = 41, red), described with a single fitted empirical function (see Methods S1, part E).

(B) Total numbers of FM B cells recovered from the same mice. The blue line shows the accrual of FM B cells from age 14 days predicted by the best-fitting model of of FM B cell dynamics in adult chimeras (Figure 4), using the time course of T1 precursors in young WT mice.

(C and D) Fits to the numbers of FM B cells in young WT mice (blue dots), using extensions of the best-fitting model in which either the rate of recruitment from the T1 pool increases with age in young mice (C), or the death rate of FM cells decreases with age (D).

(E) Predictions of the models of the normalized age distributions of FM B cells in 40-day-old mice.

(F) GFP levels among subsets of splenic B cells in 6-week-old Rag2-GFP mice.

(G) Variation in GFP expression in B cell subsets in Rag2-GFP mice, with mouse age.

(H) Fitted time dependence of the MFI of GFP among total FM B cells, in the two models (black lines). These fits were used to predict the MFI of GFP-positive cells (orange lines).

See also Figure S4.

we characterized in adults followed the same dynamics early in life. To do this, we used our best-fit time-dependent turnover model and its parameter estimates from adult mice, together with an empirical description of the changing numbers of T1 B cells in young mice (Figure 5A, solid line; Methods S1, part E) to predict the kinetics of accumulation of FM B cells from age 10 days onward, extrapolating the exponentially decaying loss rate back to the earliest time point (age 14 days). We found

that the predicted FM B cell numbers substantially overshot the observations (~3-fold higher at age 4 weeks; Figure 5B).

This mismatch indicated that either (1) cells flow from T1 to the FM B cell pool at a lower *per capita* rate early in life and/or (2) FM B cells in young mice are lost much more rapidly than those in adult mice, at even greater levels than predicted by the best-fitting model of age-dependent loss. We tested these two hypotheses by expressing both as models, allowing the *per capita* rate of influx from T1 to increase progressively with host age, or augmenting the death rate of FM B cells early in life (Methods S1, part E). We then fitted these two models to the FM B cell counts in young mice (Figures 5C and 5D; see Figure S4 for the parameter estimates). Both of these extensions described the data well, but they made distinct predictions regarding the age distribution of FM B cells in young mice (Figure 5E; Methods S1, part E). Increasing recruitment from T1 predicted a broad distribution of cell ages, while higher loss rates in young mice predicted a preponderance of younger cells. To test these predictions, we analyzed FM B cell development in Rag2-GFP transgenic mice. In these mice, GFP expression is induced in BM progenitors during RAG-mediated BCR recombination and persists into peripheral transitional and mature FM B cell populations (Figure 5F). We could then use the distribution of GFP expression within a population as a surrogate of its age distribution. As expected, GFP levels in the T1 and T2/3 compartments, which turn over rapidly, were uniformly high and did not vary with host age (Figure 5G). Average GFP fluorescence in FM B cells was high in young mice and as expected declined with age, as mature GFP-negative cells accumulated. Significantly, however, the average GFP expression in GFP-positive FM B cells, which are newly generated, was also invariant with host age. If newly generated FM B cells were shorter lived in neonates, we would expect a relative enrichment of GFP^{hi} FM B cells in younger mice, with an associated higher population-average GFP expression than in adults. This was not observed. To confirm this semiquantitative argument, we tested each model's ability to reproduce these fluorescence profiles. By directly mapping cell age to GFP fluorescence (Methods S1, part E), we fitted the age-structured formulation of each model to the time course of the mean fluorescence intensity (MFI) of GFP in total FM B cells (Figure 5H, black points). This involved estimating only two additional parameters—the GFP decay rate, and its fluorescence intensity in newly generated FM B cells. We then used each model to predict the time course of the MFI of GFP-positive FM B cells. The model of time-varying influx clearly described the data better (Figure 5H, orange lines). We infer that the relatively low rate of accumulation of FM B cells in neonates most likely derives largely from a lower rate of differentiation of T1 progenitor cells early in life, rather than shorter lifespans of FM B cells.

GC B Cells in Spleen and Lymph Nodes Exhibit Distinct Dynamics

We next applied a similar modeling approach to examine the dynamics of naturally occurring GC reactions in naive mice throughout life. Although the stimuli that drive formation of these GC reactions have not been characterized, analysis of germ-free mice revealed similar numbers of GC B cells in spleen but reduced numbers in LNs compared to WT controls in conventional facilities (Figure S5). These observations indicate that naturally occurring GC reactions are driven by self/endogenous stimuli in the spleen and a more dominant foreign source of antigen in LNs.

The number of GC B cells in the spleen gradually increased with age (Figure 6A), implying either a gradual increase in the rate of influx from their precursors, and/or increases in GC B cell lifespan or proliferation rate with age. The chimerism of splenic GC B cells stabilized within ~100 days (Figure 6B), earlier than FM B cells (~150 days, Figure 4C). This asynchrony in development discounts FM B cells as the precursors of splenic GC B cells. Therefore, we inferred that splenic GC B cells derive directly from immature transitional B cell subsets. We then fitted the models illustrated in Figure 4A to the time courses of numbers, chimerism, and Ki67 expression of splenic GC B cells. However, all of the models received comparable levels of statistical support (Table S2, rows shaded in gray) preventing us from clearly discriminating between them.

This uncertainty stems from the relatively noisy approach to stable chimerism among GC B cells, which rather poorly constrains their net loss rate, and the nearly saturating and constant levels of expression of Ki67 among host and donor cells (Figure 6C). These high levels provide relatively little information regarding the contributions of inheritance of Ki67 from the source and the division of GC B cells themselves. To increase our ability to discriminate between models, we exploited this high level of Ki67 expression. We generated a fate-mapping mouse strain in which an inducible CreERT2 construct was expressed from the endogenous *Mki67* locus, alongside a Ki67-Cherry fusion protein. Crossing these with Rosa26^{RYFP} Cre reporter mice generated a strain in which, following induction of Cre activity by the inducer tamoxifen, dividing cells and their progeny could be indelibly labeled by expression of YFP. Treating these Ki67 reporter mice with tamoxifen for just 4 days resulted in labeling of a substantial and similar fraction of GC B cells in both spleen and LNs, which declined 8 weeks after induction (Figure 6D). The fold reduction in YFP expression allowed us to place a tighter prior on the net loss rate of GC B cells (Methods S1, part F). Re-fitting the models using this information then revealed the strongest support for the model of splenic GC as a kinetically homogeneous population, fed by T2 B cells at a rate that increases gradually with mouse age (57% of the model weights, Table S2; fits shown in Figures 6A–6C). Fits using the alternative models are in Figure S6, and associated parameter estimates are in Data S1.

As expected, we inferred that splenic GC B cells are more dynamic than FM B cells, with a mean cell lifetime and mean interdivision time both roughly 12 h. Remarkably, the net effect of these tightly balanced processes yields a mean clonal lifespan of about 30 days (Table 1); thus, B cell lineages in GC are preserved for several weeks with very rapid turnover of their constituent cells. Because the total number of splenic GC B cells does not change rapidly with age in adult mice, the population can be considered close to equilibrium, and the clonal lifespan determines of the timescale of replacement of host with donor cells (Methods S1, part C). We estimate that the number of cells entering the splenic GC population per day is 4%–5% of the pool size in adulthood, a fraction that is indeed close to the

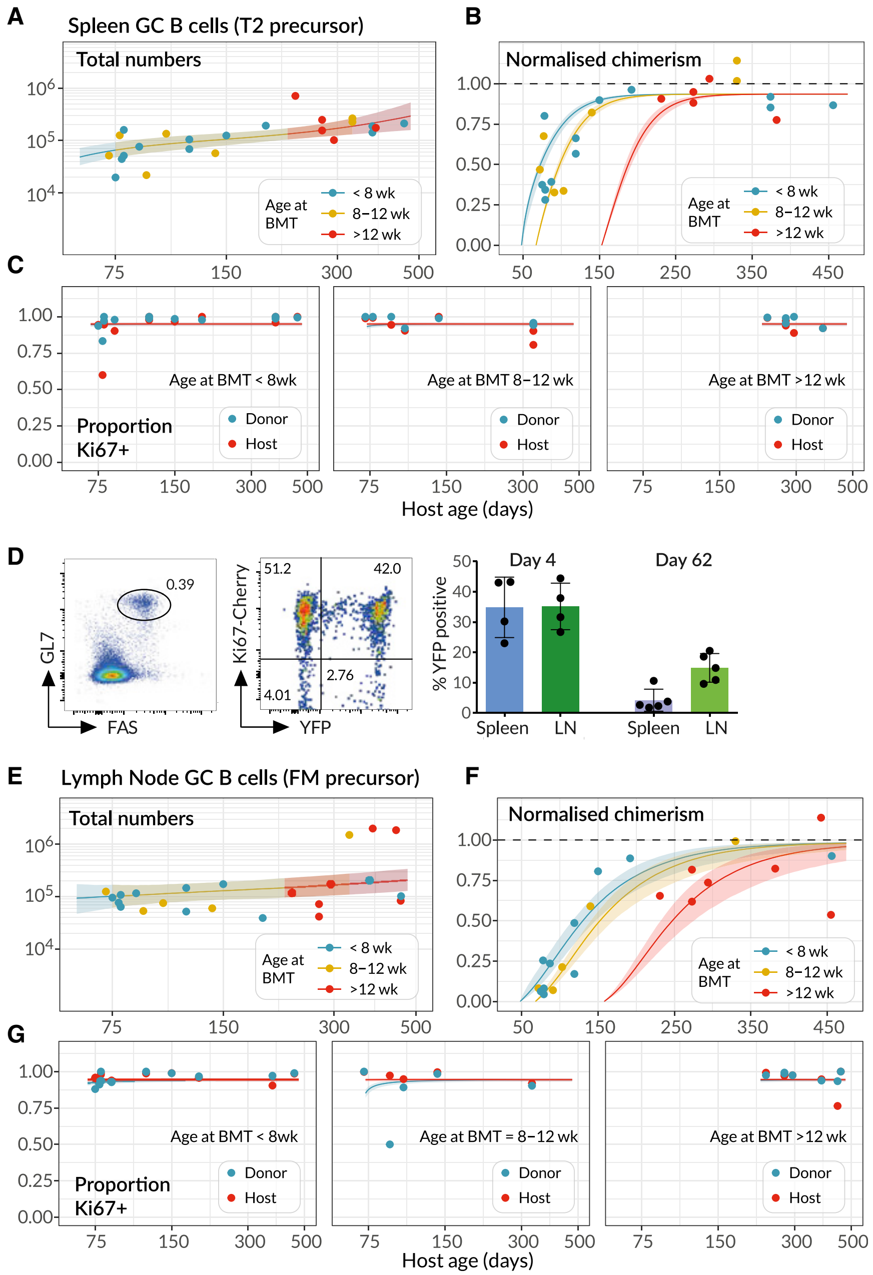

Figure 6. Modeling the Population Dynamics of Germinal Center B Cells in Spleen and Lymph Nodes

(A–C) Splenic GC B cell dynamics, with solid lines denoting the best fits to total cell counts, normalized chimerism and Ki67 expression stratified by host and donor cells. Shaded regions are prediction intervals. described in Figure 4. Line colors indicate grouping by age at BMT; <8 weeks (n = 12), 8–12 weeks (n = 7), and >12 weeks (n = 5). Curves were generated using the mean age at BMT within each group.

(D) Using the Ki67-YFP reporter mouse to track cohorts of divided cells entering the GC pools in spleen and lymph nodes. (Left panel) Gating strategy for GC B cells. (Middle panel) Cells divided during tamoxifen administration express Ki67-Cherry transiently and YFP heritably. (Right panel) Frequencies of YFP^{+} cells among GC B cells decrease with time as they are diluted by YFP^{-} immigrant cells. Data are pooled from two independent experiments.

(E–G) Analogous fits to the dynamics of lymph-node GC (LNGC) B cells, again using mice of different ages at BMT; <8 weeks (n = 11), 8–12 weeks (n = 5), and >12 weeks (n = 7).

proportion lost each day, which is approximately the inverse of the clonal lifespan.

We performed a similar analysis of LN GC (LNGC) B cell dynamics, again using the Ki67 reporter mice to place bounds on the average net loss rate of these cells and to increase our ability to distinguish between models. We found strongest support for a model of two subsets of LNGC B cells with distinct but rapid kinetics, with FM B cells as the direct precursor of both (84% relative weight, Table S3, unshaded rows; model fits shown in Figures 6E–6G), with lower support for a T2 precursor (11% relative weight, Table S3). However, the existence of this heterogeneity, and the visual similarity of the fits deriving from the two differentiation pathways (Figure S7), suggests that LNGC B cells may in fact be fed to different degrees by both FM and T2 cells.

Parameter estimates are detailed in Table 1. We estimated a low rate of influx into the LNGC pool (at most 2% of the pool size per day), with a transient subset consisting of short-lived clones (lifetime ~20 days) and a persistent subset with a clonal lifetime of ~140 days. The persistent clones comprise approximately 80% of LNGC B cells, and their long lifespan is the key determinant of the slow rate at which donor cells infiltrate the compartment (Figure 6F).

DISCUSSION

Our analysis of naive (FM) B cell homeostasis revealed continuous replenishment from the BM throughout life, with the entire compartment subject to replacement within 200 days, and the best-fitting model of the dynamics of this compartment was one in which all naive FM B cells follow the same rules of replacement irrespective of their residence history. Although it appears that all naive B cells are made and remain equal with respect to homeostatic fitness, we did find strong evidence that age-dependent changes in the host environment influenced both FM B cell maintenance and development. The most strongly supported model, in which turnover (loss) decreases with time, indicated that B cells live almost twice as long in aged mice. We also found that development of new B cells from transitional precursors is relatively

inefficient in neonates, despite an abundance of T1 and T2 precursors. It therefore appears that the host environment is the biggest factor influencing B cell homeostasis, rather than cell-intrinsic changes. Age-associated B cells (AABCs) are a subpopulation of IgM^{hi} CD23^{lo} AA4.1^{-} FAS^{+} B cells that appear in aging hosts (Naradikian et al., 2016). We observed AABCs in aged busulfan chimeras and found they were predominantly of donor origin (Figure S8), indicating that they are generated by newer cells later in life. This observation suggests that their emergence is also associated with a changing host environment and not from a cell-intrinsic increase in the propensity of aging cells to differentiate. In the latter case, one would expect a larger representation of host-derived cells within that population.

We also assessed the contribution of cell division to peripheral B cell homeostasis, through analysis of Ki67 expression. The extensive proliferation of GC B cells is a well-recognized feature of their development, but the role of cell division in supporting naive B cell homeostasis is less clear. The persistence of Ki67 for a few days following mitosis complicates the interpretation of its expression among differentiating populations, particularly when differentiation occurs over similar or shorter timescales. Our analysis revealed that Ki67 levels in peripheral naive B cells and transitional populations derive almost exclusively from proliferating BM precursors and not from proliferative self-renewal. Indeed, the best fitting model, that allowed for inheritance of Ki67 from precursors, indicated that FM B cells divide extremely rarely, if at all.

GCs are readily detectable in laboratory mice, even in the absence of deliberate infection. Although the range of stimuli that elicit such responses has not been fully characterized, commensal organisms in the gut may provide a significant antigenic drive (Reboldi and Cyster, 2016). The constant flux of new cells into these structures that we observe is consistent with the observation that new FM B cells are continually recruited into chronic GC reactions (Schwickert et al., 2007; Shulman et al., 2013) or into the same GC after repeated immunizations (Bergqvist et al., 2013). The detailed dynamics of GC reactions over short timescales, typically those of acute infections, have been modeled extensively (Kepler and Perelson, 1993; Figge, 2005; Anderson et al. 2009; Meyer-Hermann et al. 2012; Robert et al. 2017), but here we focused on GC B cell production and turnover over timescales of months to years. We revealed that GC B cell lineages—which we loosely referred to here as clones, belying the process of affinity maturation—persist for many weeks. Strikingly, we were still able to resolve the remarkably rapid cellular dynamics underlying this persistence. Our inference that the average lifespans of GC B cells are very short is consistent with observations of the frequency of apoptotic cells, which led to the conclusion that least 3% of GC B cells die per hour (Wittenbrink et al., 2011). This figure translates to minimal death rate of 0.73/day or an upper bound on the mean lifespan of 1.4 days, consistent with our estimates of 12–18 h. Our estimates of interdivision times of roughly half a day are also comparable to other estimates derived from BrdU labeling (Anderson et al., 2009). The fine balance between these two rapid processes underpins the extended lifetimes of GC B cell lineages that we expose here.

Our analyses also made distinct predictions regarding GC reactions in spleen and LNs. Splenic GC appeared to involve homogeneous dynamics, sourced primarily from T2/3 cells, although the data were sufficiently noisy to perhaps obscure any kinetic substructure. In contrast, we found evidence of heterogeneous kinetic substructures within GC reactions in LNs, which are fed predominantly from mature FM B cells. These distinct dynamics, particularly with regard to the influx of new cells, suggest differences in the nature of antigen exposure in these organs. Indeed, GC B cells were readily detectable in the spleens of germ-free mice while LN GC B cells were substantially reduced in number (Figure S5). We speculate that splenic GC B cell clones are generated predominantly by weak responses to self-antigens, and that these reactions are fed at a relatively high rate by new B cells almost as soon as they emerge from development. In contrast, LN GC B cell clones are likely derived from stronger and rarer cognate reactions of FM B cells to foreign antigens draining from epithelial barriers. Notably, the subpopulations we identified in constitutive LN reactions were both highly proliferative and so probably do not correspond to the kinetically distinct populations of B cells found in light and dark zones within GCs (Mesin et al., 2016; Meyer-Hermann et al., 2012). This heterogeneity could arise from multiple sources. GC reactions could be seeded from both FM and T2 sources, and they could also involve both newly stimulated naive B cells and recirculating memory cells, which may exhibit different kinetics. Another possibility is that, rather than representing subpopulations within the same GC, the heterogeneity we detected among LN GC B cells derives in part from the pooling of multiple LNs in our analysis, and indicates that different lymphoid organs exhibit different rates of GC B cell turnover.

We found little statistical support for models in which transitional T2 B cells are the direct precursors of FM B cells, which is perhaps surprising as they represent a more advanced stage of development than T1 cells. Notably, we found a relatively weak correlation between the chimerism of T2 cells in spleen and LNs within the same mouse (Figure 2A), suggesting that T2 cells are a spatially heterogeneous population. This result suggests that a more refined modeling approach would account for the circulation of transitional and mature B cell populations between the spleen and secondary lymphoid organs, at the cost of having to estimate a greater number of free parameters.

Our study reveals the importance of continual influx to the maintenance of naive B cell compartments in mice, which bears similarities to the mode of maintenance of naive T cells in adult mice (Hogan et al., 2015; Rane et al., 2018). In adulthood, both naive B and T cells have lifespans of several weeks and are reliant upon a daily influx of new cells that is a few percent of the pool size. We also find evidence for increased longevity of both populations as the mouse ages, that compensates to some degree for the waning of transitional B cell precursors in young adulthood, and the more substantial and longer-term involution of the thymus. The mechanisms that enhance lifespan, however, contrast between the two lymphocyte lineages. T cells exhibit cell-intrinsic adaptations such that older cells become fitter as they age and are preferentially retained in the repertoire (Rane et al., 2018), while we infer that increased B cell longevity is achieved by changes in the host that impact B cell populations uniformly. Consequently, B cells retain their homogeneous homeostatic properties, while T cell

compartments become increasingly heterogeneous with age, with evidence of naive T cell clones being retained for many months and even years (Hogan et al., 2015). The cell-intrinsic adaptation of T cells may be driven by the self-MHC recognition that has been shown to be essential for their long-term survival (Martin et al., 2006). The environmental factors responsible for age-dependent changes in B cell homeostasis remain to be identified, but are important targets for future study given the profound compartment-wide influence they wield.

STAR★METHODS

Detailed methods are provided in the online version of this paper and include the following:

- KEY RESOURCES TABLE
- RESOURCE AVAILABILITY
  - Lead Contact
  - Materials Availability
  - Data and Code Availability
- EXPERIMENTAL MODEL AND SUBJECT DETAILS
- METHOD DETAILS
  - Mouse treatments
  - Flow cytometry
- QUANTIFICATION AND STATISTICAL ANALYSIS

SUPPLEMENTAL INFORMATION

Supplemental Information can be found online at https://doi.org/10.1016/j.celrep.2020.108376.

ACKNOWLEDGMENTS

The authors acknowledge financial support from the United Kingdom Medical Research Council (MR/P011225/1 to B.S.) and the United States National Institutes of Health (R01 AI093870 to A.J.Y.). We thank Fiona Powrie and the Oxford Centre for Microbiome Studies for germ-free mice.

AUTHOR CONTRIBUTIONS

Study Conception, B.S. and A.J.Y.; Experiment Supervision, B.S.; Analysis Supervision, A.J.Y.; Experiments, M.V.; Germ-free Mice, C.P.; Mathematical Modeling, Coding, and Statistical Analyses, S.R.; Manuscript – Draft, S.R., A.J.Y., and B.S. All authors approved the final version.

DECLARATION OF INTERESTS

The authors declare no competing interests.

Received: February 25, 2020
Revised: July 29, 2020
Accepted: October 21, 2020
Published: November 17, 2020

SUPPORTING CITATIONS

The following references appear in the supplemental information: Akaike, 1974, Burnham and Anderson, 2002, Vehtari et al., 2015, Vehtari et al., 2017.

REFERENCES

Akaike, H. (1974). A new look at the statistical model identification. IEEE Trans. Automat. Contr. *19*, 716–723.

Allman, D., Lindsley, R.C., DeMuth, W., Rudd, K., Shinton, S.A., and Hardy, R.R. (2001). Resolution of three nonproliferative immature splenic B cell subsets reveals multiple selection points during peripheral B cell maturation. J. Immunol. *167*, 6834–6840.

Anderson, S.M., Khalil, A., Uduman, M., Hershberg, U., Louzoun, Y., Haberman, A.M., Kleinstein, S.H., and Shlomchik, M.J. (2009). Taking advantage: high-affinity B cells in the germinal center have lower death rates, but similar rates of division, compared to low-affinity cells. J. Immunol. *183*, 7314–7325.

Basso, K., and Dalla-Favera, R. (2015). Germinal centres and B cell lymphomagenesis. Nat. Rev. Immunol. *15*, 172–184.

Bergqvist, P., Stensson, A., Hazanov, L., Holmberg, A., Mattsson, J., Mehr, R., Bemark, M., and Lycke, N.Y. (2013). Re-utilization of germinal centers in multiple Peyer's patches results in highly synchronized, oligoclonal, and affinity-matured gut IgA responses. Mucosal Immunol. *6*, 122–135.

Burnham, K.P., and Anderson, D.R. (2002). Model Selection and Multimodel Inference: A Practical Information-Theoretic Approach, Second Edition (Springer-Verlag).

De Boer, R.J., and Perelson, A.S. (2013). Quantifying T lymphocyte turnover. J. Theor. Biol. *327*, 45–87.

De Boer, R.J., Mohri, H., Ho, D.D., and Perelson, A.S. (2003). Estimating average cellular turnover from 5-bromo-2′-deoxyuridine (BrdU) measurements. Proc. Biol. Sci. *270*, 849–858.

De Silva, N.S., and Klein, U. (2015). Dynamics of B cells in germinal centres. Nat. Rev. Immunol. *15*, 137–148.

Figge, M.T. (2005). Stochastic discrete event simulation of germinal center reactions. Phys. Rev. E Stat. Nonlin. Soft Matter Phys. *71*, 051907.

Förster, I., and Rajewsky, K. (1990). The bulk of the peripheral B-cell pool in mice is stable and not rapidly renewed from the bone marrow. Proc. Natl. Acad. Sci. USA *87*, 4781–4784.

Frasca, D., Diaz, A., Romero, M., Landin, A.M., and Blomberg, B.B. (2011). Age effects on B cells and humoral immunity in humans. Ageing Res. Rev. *10*, 330–335.

Fulcher, D.A., and Basten, A. (1997). Influences on the lifespan of B cell subpopulations defined by different phenotypes. Eur. J. Immunol. *27*, 1188–1199.

Gibson, K.L., Wu, Y.-C., Barnett, Y., Duggan, O., Vaughan, R., Kondeatis, E., Nilsson, B.-O., Wikby, A., Kipling, D., and Dunn-Walters, D.K. (2009). B-cell diversity decreases in old age and is correlated with poor health status. Aging Cell *8*, 18–25.

Gossel, G., Hogan, T., Cownden, D., Seddon, B., and Yates, A.J. (2017). Memory CD4 T cell subsets are kinetically heterogeneous and replenished from naive T cells at high levels. eLife *6*, 596.

Grabstein, K.H., Waldschmidt, T.J., Finkelman, F.D., Hess, B.W., Alpert, A.R., Boiani, N.E., Namen, A.E., and Morrissey, P.J. (1993). Inhibition of murine B and T lymphopoiesis in vivo by an anti-interleukin 7 monoclonal antibody. J. Exp. Med. *178*, 257–264.

Hao, Z., and Rajewsky, K. (2001). Homeostasis of peripheral B cells in the absence of B cell influx from the bone marrow. J. Exp. Med. *194*, 1151–1164.

Hogan, T., Shuvaev, A., Commenges, D., Yates, A., Callard, R., Thiébaut, R., and Seddon, B. (2013). Clonally diverse T cell homeostasis is maintained by a common program of cell-cycle control. J. Immunol. *190*, 3985–3993.

Hogan, T., Gossel, G., Yates, A.J., and Seddon, B. (2015). Temporal fate mapping reveals age-linked heterogeneity in naive T lymphocytes in mice. Proc. Natl. Acad. Sci. USA *112*, E6917–E6926.

Hogan, T., Yates, A., and Seddon, B. (2017). Generation of busulfan chimeric mice for the analysis of T cell population dynamics. Bio-Protocol *7*, 1–8.

Hogan, T., Nowicka, M., Cownden, D., Pearson, C.F., Yates, A.J., and Seddon, B. (2019). Differential impact of self and environmental antigens on the ontogeny and maintenance of $CD4^+$ T cell memory. eLife *8*, e48901. https://doi.org/10.7554/eLife.48.

Kepler, T.B., and Perelson, A.S. (1993). Cyclic re-entry of germinal center B cells and the efficiency of affinity maturation. Immunol. Today *14*, 412–415.

Kline, G.H., Hayden, T.A., and Klinman, N.R. (1999). B cell maintenance in aged mice reflects both increased B cell longevity and decreased B cell generation. J. Immunol. *162*, 3342–3349.

Lam, K.P., Kühn, R., and Rajewsky, K. (1997). In vivo ablation of surface immunoglobulin on mature B cells by inducible gene targeting results in rapid cell death. Cell *90*, 1073–1083.

Loder, F., Mutschler, B., Ray, R.J., Paige, C.J., Sideras, P., Torres, R., Lamers, M.C., and Carsetti, R. (1999). B cell development in the spleen takes place in discrete steps and is determined by the quality of B cell receptor-derived signals. J. Exp. Med. *190*, 75–89.

Martin, B., Bécourt, C., Bienvenu, B., and Lucas, B. (2006). Self-recognition is crucial for maintaining the peripheral CD4+ T-cell pool in a nonlymphopenic environment. Blood *108*, 270–277.

Mesin, L., Ersching, J., and Victora, G.D. (2016). Germinal Center B Cell Dynamics. Immunity *45*, 471–482.

Meyer-Bahlburg, A., Andrews, S.F., Yu, K.O.A., Porcelli, S.A., and Rawlings, D.J. (2008). Characterization of a late transitional B cell population highly sensitive to BAFF-mediated homeostatic proliferation. J. Exp. Med. *205*, 155–168.

Meyer-Hermann, M., Mohr, E., Pelletier, N., Zhang, Y., Victora, G.D., and Toellner, K.-M. (2012). A theory of germinal center B cell selection, division, and exit. Cell Rep. *2*, 162–174.

Miller, I., Min, M., Yang, C., Tian, C., Gookin, S., Carter, D., and Spencer, S.L. (2018). Ki67 is a graded rather than a binary marker of proliferation versus quiescence. Cell Rep. *24*, 1105–1112.e5.

Naradikian, M.S., Hao, Y., and Cancro, M.P. (2016). Age-associated B cells: key mediators of both protective and autoreactive humoral responses. Immunol. Rev. *269*, 118–129.

Petro, J.B., Gerstein, R.M., Lowe, J., Carter, R.S., Shinners, N., and Khan, W.N. (2002). Transitional type 1 and 2 B lymphocyte subsets are differentially responsive to antigen receptor signaling. J. Biol. Chem. *277*, 48009–48019.

Pillai, S., and Cariappa, A. (2009). The follicular versus marginal zone B lymphocyte cell fate decision. Nat. Rev. Immunol. *9*, 767–777.

Rane, S., Hogan, T., Seddon, B., and Yates, A.J. (2018). Age is not just a number: Naive T cells increase their ability to persist in the circulation over time. PLoS Biol. *16*, e2003949.

Reboldi, A., and Cyster, J.G. (2016). Peyer's patches: organizing B-cell responses at the intestinal frontier. Immunol. Rev. *271*, 230–245.

Robert, P.A., Rastogi, A., Binder, S.C., and Meyer-Hermann, M. (2017). How to Simulate a Germinal Center. Methods Mol. Biol. *1623*, 303–334.

Schwickert, T.A., Lindquist, R.L., Shakhar, G., Livshits, G., Skokos, D., Kosco-Vilbois, M.H., Dustin, M.L., and Nussenzweig, M.C. (2007). In vivo imaging of germinal centres reveals a dynamic open structure. Nature *446*, 83–87.

Shahaf, G., Johnson, K., and Mehr, R. (2006). B cell development in aging mice: lessons from mathematical modeling. Int. Immunol. *18*, 31–39.

Shulman, Z., Gitlin, A.D., Targ, S., Jankovic, M., Pasqual, G., Nussenzweig, M.C., and Victora, G.D. (2013). T follicular helper cell dynamics in germinal centers. Science *341*, 673–677.

Srinivas, S., Watanabe, T., Lin, C.S., William, C.M., Tanabe, Y., Jessell, T.M., and Costantini, F. (2001). Cre reporter strains produced by targeted insertion of EYFP and ECFP into the ROSA26 locus. BMC Dev. Biol. *1*, 4.

Srivastava, B., Quinn, W.J., 3rd, Hazard, K., Erikson, J., and Allman, D. (2005). Characterization of marginal zone B cell precursors. J. Exp. Med. *202*, 1225–1234.

Su, T.T., and Rawlings, D.J. (2002). Transitional B lymphocyte subsets operate as distinct checkpoints in murine splenic B cell development. J. Immunol. *168*, 2101–2110.

Torres, R.M., Flaswinkel, H., Reth, M., and Rajewsky, K. (1996). Aberrant B cell development and immune response in mice with a compromised BCR complex. Science *272*, 1804–1808.

Vehtari, A., Gelman, A., and Gabry, J. (2015). Efficient implementation of leave-one-out cross-validation and WAIC for evaluating fitted Bayesian models. arXiv, 1507.04544. https://arxiv.org/abs/1507.04544.

Vehtari, A., Gelman, A., and Gabry, J. (2017). Practical Bayesian model evaluation using leave-one-out cross-validation and WAIC. Stat. Comput. *27*, 1413–1432.

Victora, G.D., and Mesin, L. (2014). Clonal and cellular dynamics in germinal centers. Curr. Opin. Immunol. *28*, 90–96.

Wittenbrink, N., Klein, A., Weiser, A.A., Schuchhardt, J., and Or-Guil, M. (2011). Is there a typical germinal center? A large-scale immunohistological study on the cellular composition of germinal centers during the hapten-carrier-driven primary immune response in mice. J. Immunol. *187*, 6185–6196.

Yu, W., Nagaoka, H., Jankovic, M., Misulovin, Z., Suh, H., Rolink, A., Melchers, F., Meffre, E., and Nussenzweig, M.C. (1999). Continued RAG expression in late stages of B cell development and no apparent re-induction after immunization. Nature *400*, 682–687.

STAR★METHODS

KEY RESOURCES TABLE

REAGENT or RESOURCE	SOURCE	IDENTIFIER
Antibodies		
CD3-biotin	BD Biosciences	Cat# 553060, RRID:AB_394593
TCR-biotin	BD Biosciences	Cat# 553168, RRID:AB_394680
B220-biotin	BD Biosciences	Cat# 553085, RRID:AB_394615
B220-BV785	BioLegend	Cat# 103245, RRID:AB_11218795
B220-BV421	BioLegend	Cat# 103239, RRID:AB_10933424
CD21-PerCP-CY5.5	BioLegend	Cat# 123415, RRID:AB_1595595
CD23-FITC	BD Biosciences	Cat# 561146, RRID:AB_10611730
CD23-BUV737	BD Biosciences	Cat# 564436, RRID:AB_2738806
AA4.1-BV650	BD Biosciences	Cat# 740548, RRID:AB_2740250
CD45.1-BV650	BioLegend	Cat# 110735, RRID:AB_11124743
CD45.2-FITC	BD Biosciences	Cat# 561874, RRID:AB_10894189
CD45.2-PE-TR	BioLegend	Cat# 405725, RRID:AB_2562743
CD95-biotin	BD Biosciences	Cat# 554256, RRID:AB_395328
GL7-PerCP-Cy5.5	BioLegend	Cat# 144609, RRID:AB_2562978
IgD-BV421	BioLegend	N/A
IgM-PE-Cy7	BD Biosciences	Cat# 552867, RRID:AB_394500
Ki67-FITC	BD Biosciences	Cat# 612472, RRID:AB_399649
Ki67-PE	BD Biosciences	Cat# 561283, RRID:AB_10716060
Chemicals, Peptides, and Recombinant Proteins		
Tamoxifen	Sigma-Aldrich	Cat# T5648
Busilvex® (Busulfan)	Pierre Fabre	N/A
Critical Commercial Assays		
Streptavidin Dynabeads	Life Technologies	Cat# 11047.
Live/dead Near-IR	Life Technologies	Cat# L34976.
Streptavidin-BUV395	BD Biosciences	Cat# 564176
Streptavidin-PerCP-Cy5.5	BioLegend	Cat# 405214.
Transcription factor staining buffer set	BD Biosciences	Cat# 562574
Experimental Models: Organisms/Strains		
C57Bl6/J	UCL Comparative Biology Unit	Strain 0159
SJL.C57Bl6/J	UCL Comparative Biology Unit	Strain A423
$Mki67^{mCherry\text{-}CreERT2}$ $Rosa26^{REYFP}$	UCL Comparative Biology Unit	This study
$Rag2^{GFP}$	The Jackson Laboratory	Stock No. 005688.
Software and Algorithms		
FlowJo Software	FlowJo LLC	N/A
GraphPad Prism	GraphPad Software, Inc	N/A
Custom R code used for modeling and data analysis	https://github.com/sanketrane/B_cells_FM_GC	N/A

RESOURCE AVAILABILITY

Lead Contact

Further information and requests for experimental resources and reagents should be directed to and will be fulfilled by the Lead Contact, Benedict Seddon (benedict.seddon@ucl.ac.uk). Queries regarding mathematical and statistical analyses should be directed to Andrew Yates (andrew.yates@columbia.edu).

CellPress
OPEN ACCESS

Materials Availability

Mouse strains generated in this study are available upon request subject to MTA.

Data and Code Availability

Data S1 contains the parameter estimates for all models, and all data used in this study. In addition, all code and data used to perform model fitting, and details of the prior distributions for parameters, are available at https://github.com/sanketrane/B_cells_FM_GC.

EXPERIMENTAL MODEL AND SUBJECT DETAILS

*Mki*67$^{mCherry-CreERT2}$ mice were generated by targeted replacement of the terminal exon 14 of the *Mki67* locus with a modified exon 14 sequence with upstream FRT flanked neomycin cassette, and downstream mCherry fusion construct, IRES sequence and CreERT2 cDNA. Mice were crossed with *actin-FLPe* mice to excise the neomycin selection cassette, before crossing with the *Rosa*26^{RYFP} strain (Srinivas et al., 2001), to generate *Mki*67$^{mCherry-CreERT2}$ *Rosa*26^{RYFP} double reporter mice. Cre recombinase activity was induced *in vivo* in these mice following their i.p. injection with 2mg of tamxoxifen (Sigma) diluted in corn oil (Fisher Scientific) for five consecutive days. All mice used in this study, including Rag2^{GFP} mice (Yu et al., 1999), were female and bred at Charles River UK Ltd and the Comparative Biology Unit, Royal Free Hospital. Ki67 reporter mice were aged 8-12 weeks; busulfan chimeric mice and controls were aged as indicated in figure legends. Experiments were performed according to the UCL Animal Welfare and Ethical Review Body and Home Office regulations. Germ-free mice were housed at the Oxford Centre for Microbiome Studies, Oxford, UK.

METHOD DETAILS

Mouse treatments

Busulfan chimeras were generated as previously described (Hogan et al., 2015, 2017). C57Bl6/J mice were used as bone marrow donors and SJL.C57Bl6/J congenic mice as hosts. Host age ranges are as indicated in the figures. Donor bone marrow was obtained from femurs of age-matched C57Bl6/J mice. Thereafter, these bone marrow suspensions were depleted of T and B cells by immunomagnetic selection, using biotinylated antibodies to respectively CD3 (eBioscience, 1/500 dilution), TCR-beta (eBioscience, 1/500 dilution) and B220 (eBioscience, 1/200 dilution). Captured cells were bound to streptavidin-coupled Dynabeads (Life Technologies) and the unbound fraction depleted of mature T cells and B cells. 24 hours after the final busulfan injection, eight to ten million cells were injected i.v. in the busulfan-treated mice. At the indicated time points after BMT, host mice were sacrificed and spleen, lymph nodes and bone marrow were harvested and processed for further analysis.

Flow cytometry

Flow cytometric analyses were performed on 2×10^6 cells from organs of interest. Cells were stained for 1 hour in the dark at 4°C with monoclonal antibodies (Abs) at a saturating concentration in 100 μL of PBS. The following surface antigens were detected by the indicated mAb clone: B220 (RA3-6B2- BV785 and RA3-6B2-BV421, BioLegend), CD21 (7EG-PerCP-Cy5.5, Biolegend), CD23 (B3B4-FITC, Biolegend; B3B4-BUV737, BD Biosciences), CD45.1 (A20-BV650, Biolegend), CD45.2 (104-FITC, eBioscience; 104-PE-TR, Biolegend), CD93 (AA4.1-APC, Biolegend), CD95 (Jo2-biotin, BD Biosciences), GL7 (Ly77-PerCP-Cy5.5, Biolegend), IgD (11-26x.2a-BV421, Biolegend), IgM (Il/41-PE-Cy7, eBioscience) and live/dead Near-IR (Life Technologies). A secondary staining step was performed using streptavidin-BUV395 (BD Biosciences, 0.5 μg/ml) or streptavidin-PerCP-Cy5.5 (BioLegend, 0.4 μg/ml). Cells were stained for 30 min in the dark at 4°C. Subsequently, cells were washed in handling media, and immediately analyzed by flow cytometry. For intracellular staining, cells were fixed and permeabilised using the FoxP3/transcription factor staining buffer set (eBioscience). Ki67 was detected using SolA15-FITC or SolA15-PE (eBioscience). Unless otherwise stated, individual populations were electronically gated as; splenic T1 cells, B220^{hi} AA4.1^{pos} IgM^{hi} CD23^{low}; splenic and lymph node T2/3 cells, B220^{hi} AA4.1^{pos} CD23^{hi}; FM B cells, B220^{hi} AA4.1^{neg} CD23^{hi}; and GC B cells, B220^{hi} GL7^{hi}. Data were analyzed using FlowJo v10 (Becton Dickinson & Company).

QUANTIFICATION AND STATISTICAL ANALYSIS

For pooled FM B cells, splenic GC B cells and lymph node GC B cells we fitted each mathematical model – schematically illustrated in Figure 4A – simultaneously to the time courses of total cell numbers, normalized chimerism, and Ki67 expression in host and donor cells, using empirical descriptions of the time courses of their putative precursor populations (Methods S1, Part A). In Methods S1, Part B we detail the mathematical models describing these dynamics. In Methods S1, Part C we show how the rate of percolation of donor cells into each B cell subpopulation is determined primarily by the average lifespan of B cell clones within that subpopulation.

Our approach to fitting and model selection is detailed in Methods S1, Part D. Briefly, (i) we formulated the joint likelihood of the observations, then (ii) used a Bayesian estimation approach with this likelihood and prior distributions of the model parameters to generate posterior distributions of these parameters. This procedure yielded a combined measure of the model's quality of fit and its complexity (the Leave-One-Out Information Criterion, LOO-IC). Model fits in Figures 4, 5, and 6 were generated using the maximum *a posteriori* probability (MAP) estimates of the parameters, and are accompanied by envelopes that represent the spread

of model predictions generated by sampling over these posterior distributions. Narrow envelopes therefore indicate that the model predictions are robust to variation in parameters; wide envelopes indicate sensitivity to parameter values. Details of the extensions required for modeling FM B cell dynamics in young mice are given in Methods S1, Part E. Priors on the net loss rates of splenic and lymph node GC B cells were informed by data from the Ki67-YFP reporter mice, and are described in Methods S1, Part F.

Cell Reports, Volume *33*

Supplemental Information

Fate Mapping Quantifies the Dynamics of B Cell Development and Activation throughout Life

Melissa Verheijen, Sanket Rane, Claire Pearson, Andrew J. Yates, and Benedict Seddon

Supplemental Information

Figure S1: Variation in the degree of stable chimerism in B cell subsets within the same mice. Related to Figure 1.

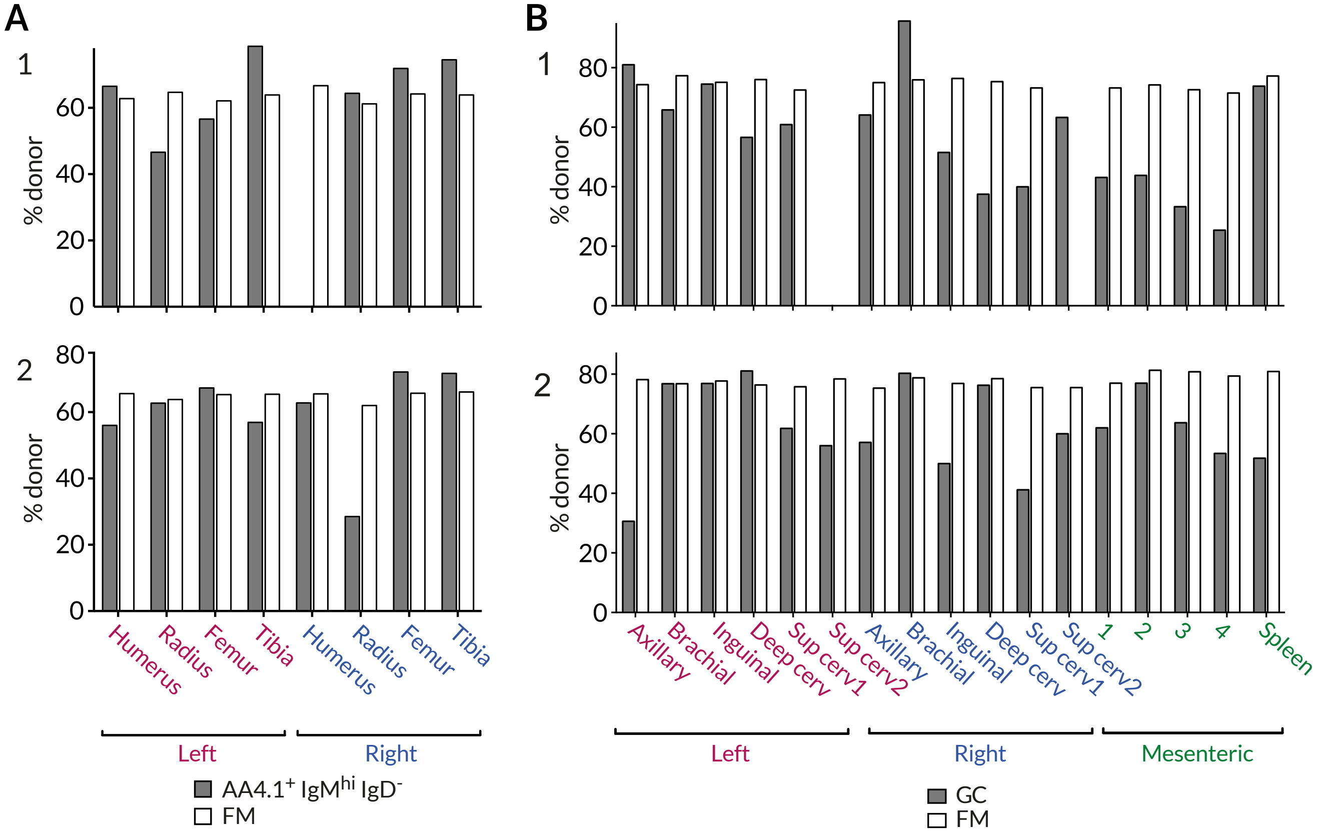

(A) Variation in the chimerism among AA4.1^{+}IgM^{hi}IgD^{-} B cell progenitors and recirculating FM B cells within different BM sites. **(B)** Variation in chimerism across different lymph nodes. Data from two representative animals.

Figure S2: Empirical descriptions of the timecourses of numbers (A-D) and chimerism (E-H) of potential B cell precursors of FM and GC B cells. Related to Figure 4.

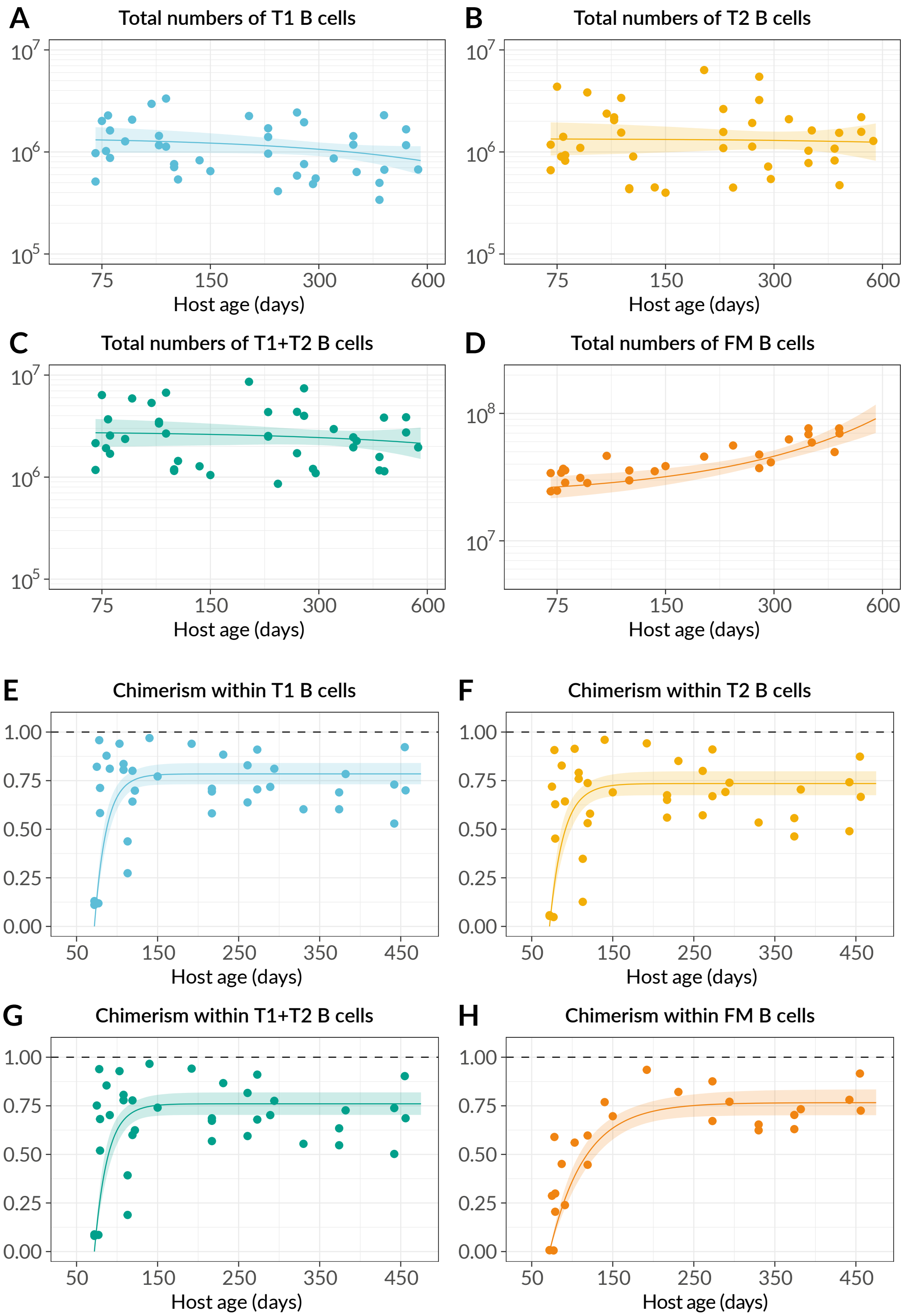

Figure S3: Fitted alternative models of FM B cell dynamics. Related to Figure 4.

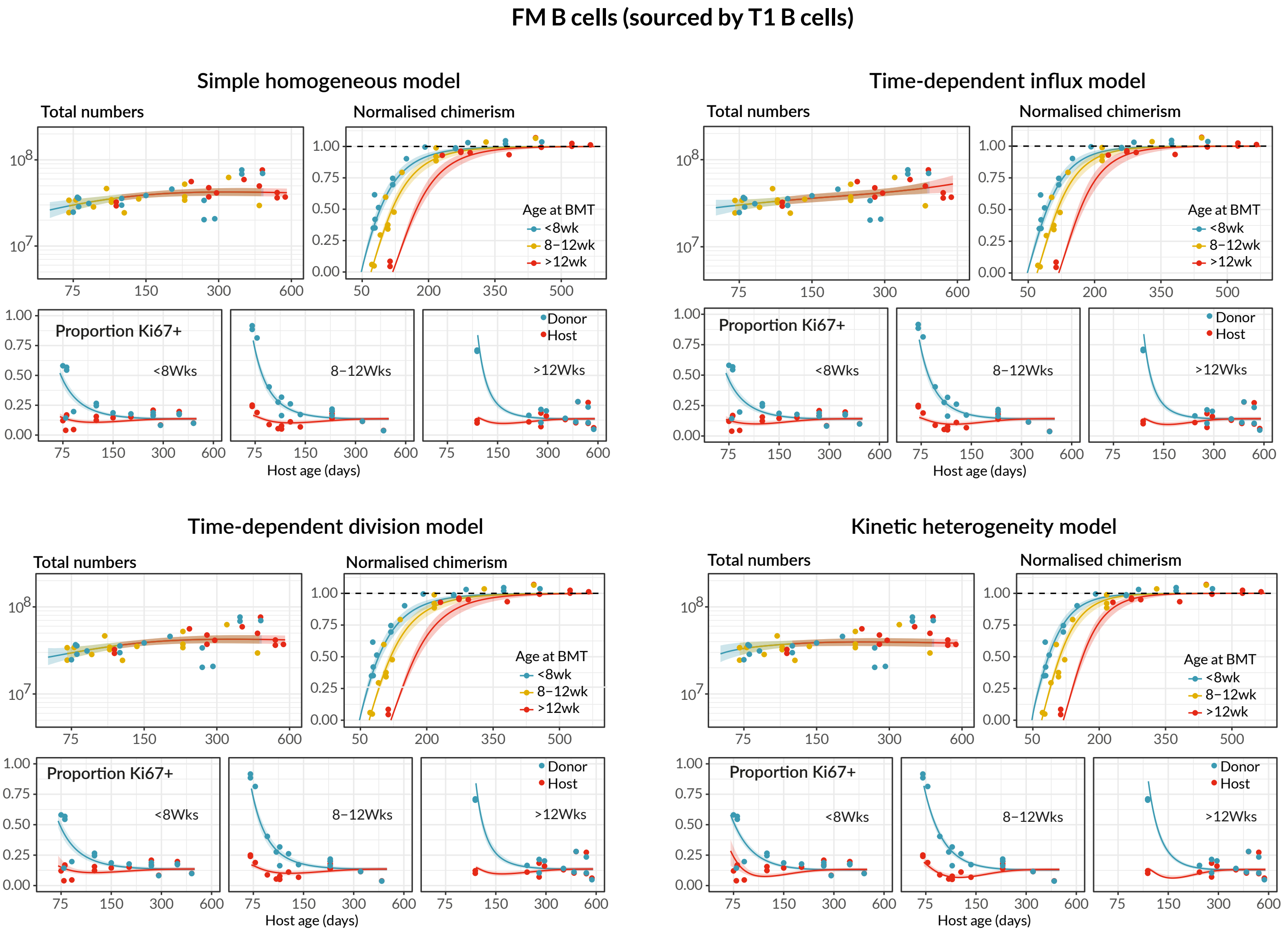

Figure S4: Predictions from competing models of FM B cell dynamics in young mice. Related to Figure 5.

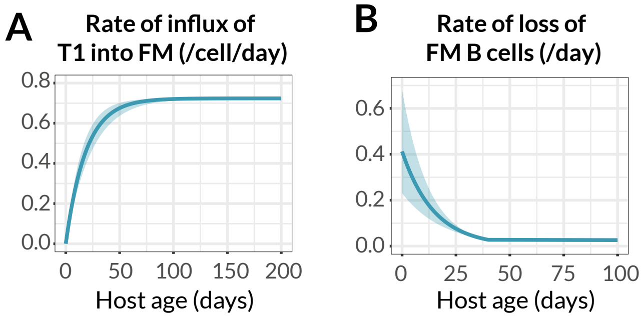

(A) Best fitting functional form for the change in *per capita* daily rate of influx from T1 to FM. **(B)** Best fitting functional form for the change in loss rate of FM with age.

Figure S5: Comparing germinal centre B cell numbers in germ free, busulfan chimeric and WT mice. Related to Figure 6.

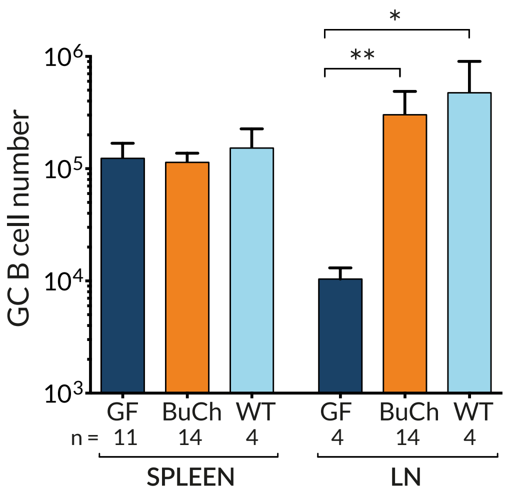

Absolute numbers of GC B cells in spleen and total LN from germ free (GF), busulfan chimeras (BuCh), and WT controls from a conventional barrier facility. Groups were of ages 10 weeks, 10-20 weeks and 10-14 weeks respectively. * $p<0.05$, ** $p<0.01$.

Figure S6: Fitted alternative models of splenic germinal centre B cell dynamics. Related to Figure 6.

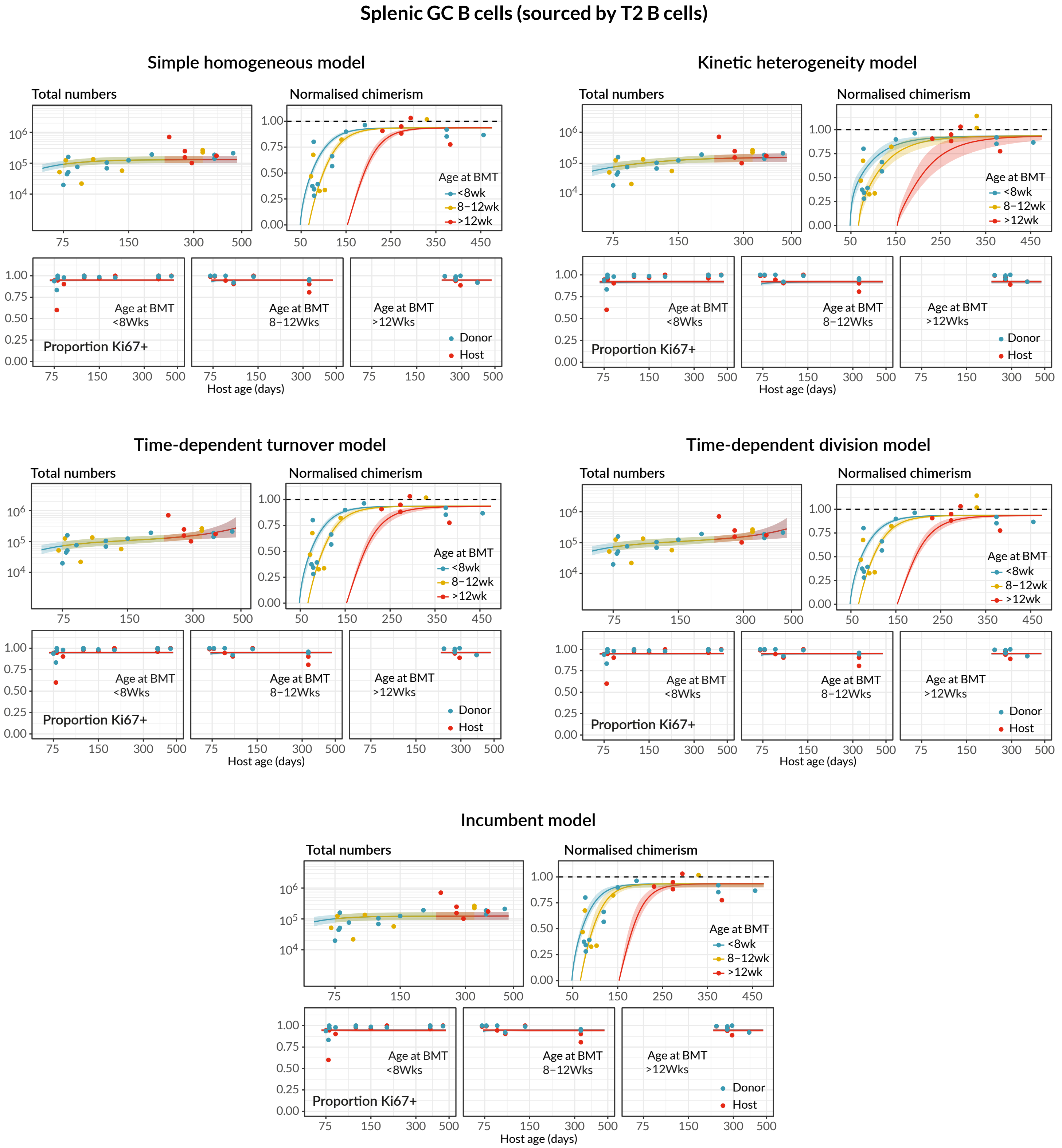

Figure S7: Fitted alternative models of lymph node germinal centre B cell dynamics. Related to Figure 6.

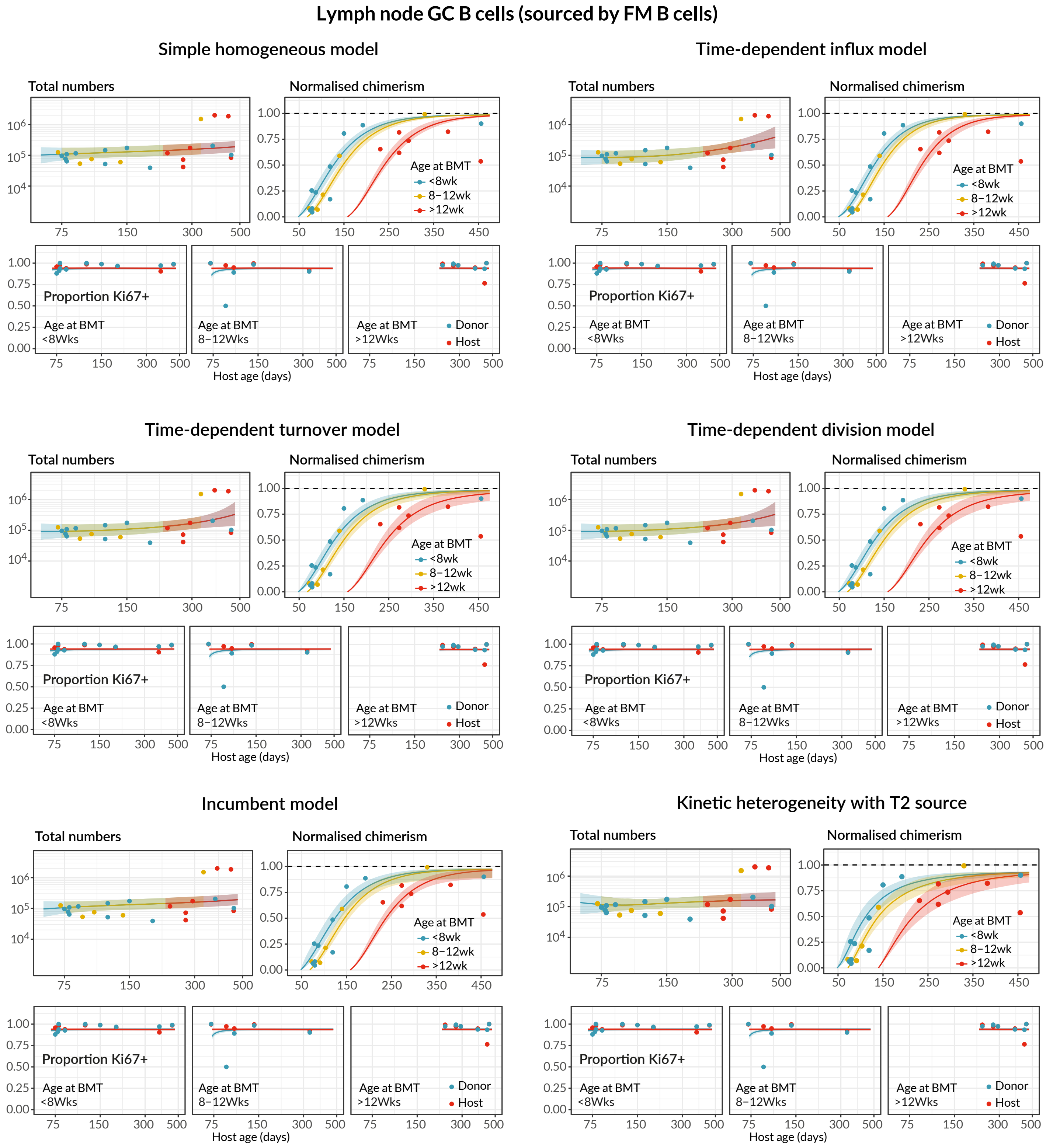

Figure S8: Age-associated B cells (AABC) accumulate in older mice. Related to Figure 4.

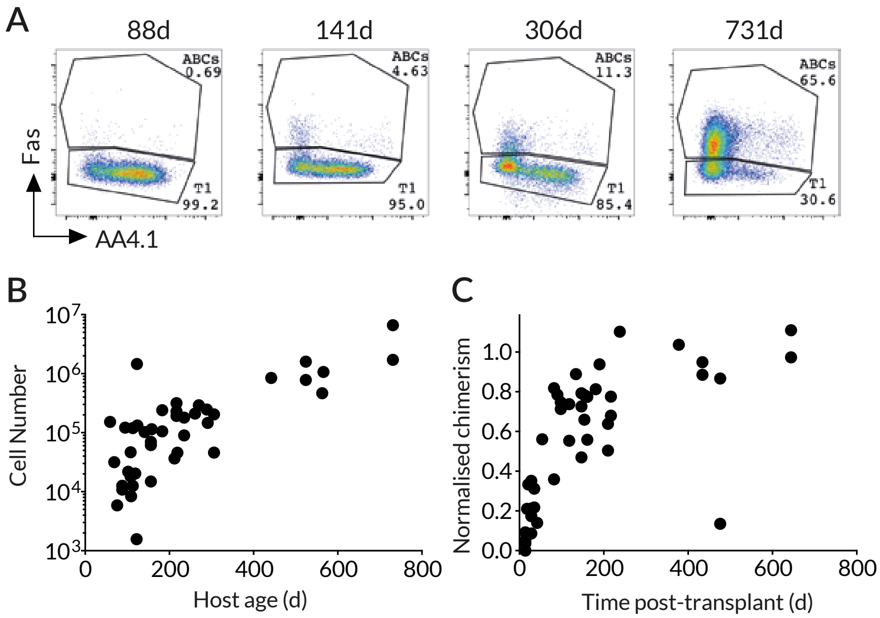

(**A**) Gating strategy for AABC. (**B**) AABC increase in number with host age. (**C**) AABC turn over within ∼300 days.

Table S1: Comparison of models describing the population dynamics of Follicular Mature (FM) B cells, pooled from LN and spleen, using Akaike weights (Methods S1, part D) as percentage measures of relative support. The most strongly favoured model is highlighted. Related to Figure 4.

**Source**	**Model and Akaike weight (%)**				
	Simple homogeneous	Time-dependent turnover	Time-dependent division	Time-dependent influx	Kinetic heterogeneity
**T1**	9.0	**64**	3.0	18	5.0
**T2**	0.0	0.0	0.0	0.0	0.0
**T1 + T2**	0.0	0.5	0.0	0.5	0.0

Table S2: Comparison of models describing the population dynamics of germinal centre (GC) B cells in spleen, with or without the additional information regarding proliferation from the Ki67 reporter mice. *Showed weak time-dependence in the rates of turnover (halving every ∼800 mo) or division (doubling every >1000 mo); were rejected in favour of the simple homogeneous model. Related to Figure 6.

**Source**	**Model and Akaike weight (%)**					
	Simple homogeneous	Time-dependent turnover	Time-dependent division	Time-dependent influx	Kinetic heterogeneity	Incumbent
Without informing models using data from Ki67-Cre-ER^{T2}-YFP reporter mice						
T1	18	16	7	12	5	18
T2	5	11	1	2	1	4
Informing models using data from Ki67-Cre-ER^{T2}-YFP reporter mice						
T1	0	3	2	9	0	0
T2	2	16*	11*	**57**	0	0

Table S3: Comparison of models describing the population dynamics of GC B cells in lymph nodes. Related to Figure 6.

**Source**	**Model and Akaike weight (%)**					
	Simple homogeneous	Time-dependent influx	Time-dependent turnover	Time-dependent division	Kinetic heterogeneity	Incumbent
Without informing models using data from Ki67-Cre-ER^{T2}-YFP reporter mice						
T1	0	21	1	0	0	0
T2	0	22	0	0	0	0
FM	6	37	11	2	0	0
Informing models using data from Ki67-Cre-ER^{T2}-YFP reporter mice						
T1	0	0	0	0	5	0
T2	0	0	0	0	11	0
FM	0	0	0	0	**84**	0

Methods S1

Details of all mathematical and statistical analyses; related to Figures 4-6.

A – Modelling the dynamics of precursor cell numbers and chimerism

We considered T1, T2, and T1+T2 combined as the potential direct precursors (sources) of FM B cells, and T1, T2 and FM B cells as potential sources of GC B cells. In adult mice, we described the time-variation in the sizes of these populations with the empirical descriptor function $S(t) = S_0\, e^{-\nu\, t}$ (Fig. **??**, panels A-D), where the parameters S_0 and ν were estimated by fitting to the log-transformed cell counts using least squares.

Similarly, the timecourses of donor chimerism in these populations were all described well with $\chi(t) = \chi_{\text{stable}}\,(1 - e^{-\nu\, t})$, shown in Fig. **??**, panels E-H; here, χ_{stable} and ν were estimated using non-linear least squares.

We assumed a constant *per capita* rate of influx ψ from the source $S(t)$, giving a total influx of $\phi(t) = \psi S(t)$ cells/day. The daily influx of host and donor cells into the target population is then

$$\begin{aligned} \phi_{\text{donor}}(t) &= \psi\, S(t)\, \chi(t), \\ \phi_{\text{host}}(t) &= \phi(t) - \phi_{\text{donor}}(t). \end{aligned} \tag{S1}$$

The unknown ψ is estimated along with the other model parameters. In the time-dependent recruitment model, we assumed the form $\psi(t) = \psi_0\, e^{p\, t}$, and estimated ψ_0 and p.

B – Mathematical models

Simple homogeneous model: In this model we assume that cells form a kinetically homogeneous population that self-renews through homeostatic division with first-order kinetics at rate α, and is lost (turns over) at a rate δ, which combines death and onward differentiation. The inverse of α is the mean interdivision time, and the inverse of δ is the mean residence time of a cell. Influx of cells from the source compartment is denoted $\phi(t)$, which is the product of the *per capita* rate of influx ψ and the timecourse of the size of precursor population $S(t)$, which is described empirically (see part A). We model the dynamics of Ki67^{hi} (H) and Ki67^{lo} (L) cells using the following ODE model;

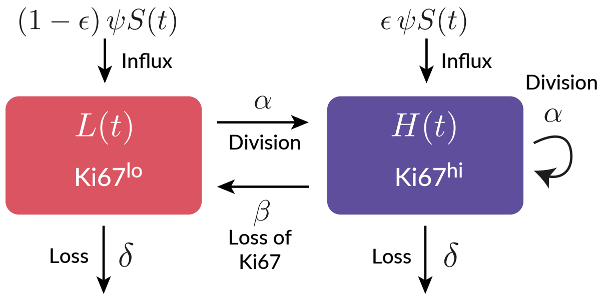

$$\begin{aligned} \dot{L}(t) &= \psi S(t)(1-\epsilon)\ + \beta\, H(t) - (\alpha + \delta)\, L(t), \\ \dot{H}(t) &= \psi S(t)\epsilon\ + \alpha\,(2\, L(t) + H(t)) - (\beta + \delta)\, H(t). \end{aligned} \tag{S2}$$

Here, β is the rate of loss of Ki67 expression after mitosis, and ϵ is the proportion of the cells entering from the source that are Ki67^{hi}; we used its average observed value in the model fitting process. We assumed eqns. S2 held identically for host and donor cells. We fitted the following combinations of the solutions to these equations simultaneously to

the timecourses of

$$\begin{aligned}
\text{Total cell numbers} &= N(t) = H_{\text{donor}}(t) + H_{\text{host}}(t) + L_{\text{donor}}(t) + L_{\text{host}}(t) \\
\text{Normalised chimerism} &= \frac{1}{\chi_{\text{source}}(t)} \frac{H_{\text{donor}}(t) + L_{\text{donor}}(t)}{N(t)} \\
\text{Proportions of donor and host cells expressing Ki67} &= \frac{H_{\text{donor}}(t)}{H_{\text{donor}}(t) + L_{\text{donor}}(t)}, \quad \frac{H_{\text{host}}(t)}{H_{\text{host}}(t) + L_{\text{host}}(t)},
\end{aligned}$$

using the empirical descriptions of the size ($S(t)$) and chimerism ($\chi_{\text{source}}(t)$) of the source population (see part A). We define time t_0 to be age at BMT of the youngest recipient (approximately 7 weeks), at which time the size of donor compartment is zero. Therefore, the Ki67^{hi} proportion among donor cells at t_0 reflects that in the source, $\kappa_{\text{donor}}(t_0) = \epsilon$. The Ki67^{hi} proportion among host cells at t_0 is defined as κ_0. We estimated β, α, δ, ψ, κ_0 and $H(t_0) + L(t_0)$, the size of the host compartment at t_0.

Time-dependent models: In these extensions of the model above, the *per capita* rate of influx of new cells from the source population ψ, the rate of cell division α, or the rate of loss δ may vary with time. We allowed each sub-model to exhibit time-dependence in only one process. These three sub-models are also homogeneous; at any given instant, all cells in the population exhibit the same rates of division and turnover. We assumed that the time-dependent parameter varied with mouse age t as $\exp(rt)$, where r was estimated from the data and was unconstrained (*i.e.* the rate constant could either rise or fall with time).

Kinetic-heterogeneity model: This model comprises two subsets, which are independent, fed separately from the same source population, and are lost and/or divide at different rates. The resulting dynamics of the population as a whole are therefore the weighted average of the more 'transient' subset (rapid net loss, $\delta_f - \alpha_f$) and a 'persistent' subset (slower net loss, $\delta_s - \alpha_s$). We solve the following equations for Ki67^{hi} and Ki67^{lo} cells among the transient and persistent subsets, and formulate it identically for host and donor cells;

$$\begin{aligned}
\dot{H}_f(t) &= \phi(\psi, t)\, f\, \epsilon \, + \alpha_f \,(2\, L_f(t) + H_f(t)) - (\beta + \delta_f)\, H_f(t), \\
\dot{L}_f(t) &= \phi(\psi, t)\, f\, (1 - \epsilon) \, + \beta\, H_f(t) - (\alpha_f + \delta_f)\, L_f(t), \\
\dot{H}_s(t) &= \phi(\psi, t)\, (1 - f)\, \epsilon \, + \alpha_s \,(2\, L_s(t) + H_s(t)) - (\beta + \delta_s)\, H_s(t), \\
\dot{L}_s(t) &= \phi(\psi, t)\, (1 - f)\, (1 - \epsilon) \, + \beta\, H_s(t) - (\alpha_s + \delta_s)\, L_s(t).
\end{aligned} \tag{S3}$$

Along with the kinetic parameters we also estimate the proportions of the transient subset in the precursor (f) and in the target (q) populations, and the initial fractions of Ki67^{hi} cells in the transient and persistent subsets. The initial numbers of host-derived cells in the transient and persistent subsets are defined as $N_0\, q$ and $N_0\, (1 - q)$, respectively.

Incumbent model: In this model, described in Hogan et al. (2015) and Rane et al. (2018), heterogeneity is exhibited only in the host compartment, which is assumed to comprise (i) an 'incumbent' subset of older, self-renewing cells that are resistant to displacement by new cells and (ii) a 'displaceable' subset that is replaced continuously by cohorts of new cells entering the pool. All donor cells are assumed to behave as displaceable cells.

$$\begin{aligned}
\dot{H}(t) &= \phi(\psi, t)\epsilon \, + \alpha\,(2\, L(t) + H(t)) - (\beta + \delta)\, H(t), \\
\dot{L}(t) &= \phi(\psi, t)(1 - \epsilon) \, + \beta\, H(t) - (\alpha + \delta)\, L(t), \\
\dot{H}_{\text{inc}}(t) &= \alpha_{\text{inc}}\,(2\, L_{\text{inc}}(t) + Y_{\text{inc}}(t)) - (\beta + \delta_{\text{inc}})\, H_{\text{inc}}(t), \\
\dot{L}_{\text{inc}}(t) &= \beta\, H_{\text{inc}}(t) - (\alpha_{\text{inc}} + \delta_{\text{inc}})\, L_{\text{inc}}(t).
\end{aligned} \tag{S4}$$

We assume that the incumbent subset is established early in life, before the minimum age of BMT in our chimeric animals ($\sim$7 weeks).

C – The time taken to approach to stable chimerism in a B cell population is determined predominantly by the clonal lifetime

Here we illustrate for the simplest homogenous model the factors that determine the rate at which chimerism in a population reaches that of its precursor population. Assume a population $N(t)$ is fed by precursors at constant total rate ϕ, divides at *per capita* rate α and is lost through death or differentiation at *per capita* rate δ;

$$dN/dt = \phi - (\delta - \alpha)N(t).$$

The quantity $\delta - \alpha$ is the net loss rate, which we denote λ:

$$dN/dt = \phi - \lambda N(t).$$

Assume the source acquires a stable chimerism χ, and that host (h) and donor (d) cells behave identically;

$$\begin{aligned} dN_d/dt &= \chi\phi - \lambda N_d(t) \\ dN_h/dt &= (1-\chi)\phi - \lambda N_h(t) \end{aligned}$$

The normalised chimerism of the population is

$$\chi_{\text{norm}} = \frac{N_d}{\chi(N_d + N_h)},$$

which evolves according to

$$\begin{aligned} \frac{d}{dt}\chi_{\text{norm}}(t) &= \frac{1}{\chi N(t)}\left(\chi\phi - \lambda N_d(t) - \frac{N_d(t)}{N(t)}\frac{dN(t)}{dt}\right) \\ &= \frac{1}{\chi N(t)}\left(\chi\phi - \lambda N_d(t) - \frac{N_d(t)}{N(t)}\left(\phi - \lambda N(t)\right)\right) \\ &= \frac{1}{\chi N(t)}\left(\chi\phi - \phi\frac{N_d(t)}{N(t)}\right) \\ &= \frac{\phi}{N(t)}\Big(1 - \chi_{\text{norm}}(t)\Big). \end{aligned} \tag{S5}$$

If the population is at equilibrium then $N(t) = \phi/\lambda$, giving

$$\frac{d}{dt}\chi_{\text{norm}}(t) = \lambda\Big(1 - \chi_{\text{norm}}(t)\Big), \tag{S6}$$

which implies

$$\chi_{\text{norm}}(t) = 1 - e^{-\lambda t}; \tag{S7}$$

that is, the chimerism in the population reaches that of its precursors at a rate determined purely by the clonal lifespan $1/\lambda$. If the population is initially out of equilibrium at size N_0,

$$\chi_{\text{norm}}(t) = 1 - \frac{1}{1 + (e^{\lambda t} - 1)\frac{\phi}{\lambda N_0}}, \tag{S8}$$

the rate of approach to $\chi_{\text{norm}} = 1$ is then governed by both λ and the daily influx as a proportion of the initial pool size, ϕ/N_0 (intuitively, if the pool is initially over-populated, $\phi/\lambda N_0 < 1$ and chimerism increases more slowly because of the excess of host cells; if the pool is depleted, $\phi/\lambda N_0 > 1$ and stable chimerism is achieved more quickly). Equation S8 reduces to S7 when $N_0 = \phi/\lambda$.

D – Fitting and selecting mathematical models

Likelihood

We attempted to explain the kinetics of host and donor cells in busulfan chimeric mice with an array of mathematical models, detailed in the main text and illustrated in Fig. 4A. As described, variation in the degree of depletion of host HSCs by busulfan treatment led to mouse-to-mouse variation in the level of stable bone-marrow chimerism (the fraction that are donor-derived), and hence also in peripheral subsets. We removed this variation by dividing the chimerism in each B cell subset by the chimerism χ in the T1 precursor population. This normalised chimerism (donor fraction) is

$$\chi_{\text{norm}} = \frac{\text{Donor cell numbers}}{\text{Total cell numbers} \times \chi}.$$

This approach allows us to fit a single model to data from multiple mice. Each model was fitted simultaneously to the timecourses of total cell counts ($N(t)$, the sum of host and donor cells), the normalised chimerism $\chi_{\text{norm}}(t)$, and the proportions of Ki67^{hi}cells in the host and donor compartments ($\kappa_{\text{host}}(t)$ and $\kappa_{\text{donor}}(t)$). Cell counts were log-transformed while χ_{norm}, κ_{host} and κ_{donor} were logit-transformed, to ensure that measurement errors were approximately normally distributed. The joint likelihood of the datasets (with variables representing their transformed values) is then

$$\begin{aligned}\mathcal{L} = \prod_{i=1}^{n} \frac{\exp\left(\frac{-(N_i - N_i^{\text{pred}})^2}{2\sigma_N^2}\right)}{\sqrt{2\pi}\sigma_N} \times \frac{\exp\left(\frac{-(\chi_{\text{norm},i} - \chi_{\text{norm},i}^{\text{pred}})^2}{2\sigma_\chi^2}\right)}{\sqrt{2\pi}\sigma_\chi} \times \frac{\exp\left(\frac{-(\kappa_{i,\text{host}} - \kappa_{i,\text{host}}^{\text{pred}})^2}{2\sigma_{\kappa_{\text{host}}}^2}\right)}{\sqrt{2\pi}\sigma_{\kappa,\text{host}}} \times \frac{\exp\left(\frac{-(\kappa_{i,\text{donor}} - \kappa_{i,\text{donor}}^{\text{pred}})^2}{2\sigma_{\kappa_{\text{donor}}}^2}\right)}{\sqrt{2\pi}\sigma_{\kappa,\text{donor}}} \\ \equiv \frac{\exp(-\text{SSR}_N/2\sigma_N^2)}{\left(\sqrt{2\pi}\sigma_N\right)^n} \times \frac{\exp(-\text{SSR}_\chi/2\sigma_\chi^2)}{\left(\sqrt{2\pi}\sigma_\chi\right)^n} \times \frac{\exp(-\text{SSR}_{\kappa,\text{host}}/2\sigma_{\kappa,\text{host}}^2)}{\left(\sqrt{2\pi}\sigma_{\kappa,\text{host}}\right)^n} \times \frac{\exp(-\text{SSR}_{\kappa,\text{donor}}/2\sigma_{\kappa,\text{donor}}^2)}{\left(\sqrt{2\pi}\sigma_{\kappa,\text{donor}}\right)^n},\end{aligned}$$

where n is the number of animals, each yielding four observations, and SSR denotes the sum of squared residuals, with each being a function of the data and the model parameters. This gives the joint log-likelihood (up to a constant);

$$\log \mathcal{L} = -\frac{1}{2}\left(\frac{\text{SSR}_N}{\sigma_N^2} + \frac{\text{SSR}_\chi}{\sigma_\chi^2} + \frac{\text{SSR}_{\kappa,\text{host}}}{\sigma_{\kappa,\text{host}}^2} + \frac{\text{SSR}_{\kappa,\text{donor}}}{\sigma_{\kappa,\text{donor}}^2}\right) - n(\log \sigma_N + \log \sigma_\chi + \log \sigma_{\kappa,\text{host}} + \log \sigma_{\kappa,\text{donor}}).$$

Parameter estimation

We used a Bayesian approach to estimating the model parameters, the errors associated with the measurements in each dataset, and a measure of support for each model. The inputs to this procedure are the joint likelihood shown above, and a set of prior distributions on the model parameters and the unknown measurement errors in each dataset. We refer to these unknowns collectively as θ. The Bayesian procedure updates these priors with the likelihood, to generate posterior distributions of θ that reflect our knowledge of these parameters in the light of the data, collectively denoted y. Strong (narrow) priors help to regularise a model's behaviour and prevent it from learning too much from the data – and hence guard against over-fitting. The joint posterior distribution of the

parameters is calculated using Bayes' rule,

$$p(\theta_{\text{post}}|y) = \frac{p(y|\theta_{\text{post}}) \cdot p(\theta_{\text{prior}})}{p(y)} \tag{S9}$$

where $p(y)$ is the likelihood of the data (averaged over the priors) that normalises the posterior such that it integrates to 1. We consider priors to be tools that improve a model's ability to learn from the data, and subjected them to similar standards of evaluation and revaluation as any other component of the model. Detailed descriptions of the priors, together with the code and data for performing all of the analyses presented in this study, are available at `github.com/sanketrane/B_cells_FM_GC`.

The models were represented as systems of ordinary differential equations (ODEs), described in detail in Methods S1, part B. We solved them numerically using the *integrate_ode_rk45* solver in the *Stan* programming language and used the default no-U-turn sampler (NUTS) to generate the posterior distributions of the parameters. We confirmed that the log-transformed cell counts, and the logit-transformed values of the normalised chimerism and the Ki67^{hi} proportions in host and donor compartments, were all normally distributed with constant errors (standard deviations). These standard deviations were additional parameters that were estimated from the data. We used the *R-stan* package in *R* to interface and compile the *Stan* scripts that encoded the priors, model definitions, and the sampling and fitting procedures.

Comparing models

The assessment of a model's utility depends on how accurately it explains a given dataset (measured by the likelihood) as well as its ability to accurately predict new observations. A complex model with an excessive number of parameters will tend to overfit any given dataset and perform poorly when predicting new observations. On the other hand, a model that is too simple will fail to capture trends in the data, generate a low likelihood, and will also make poor predictions of new observations. The Akaike Information Criterion (Akaike, 1974, Burnham and Anderson, 2002) is commonly used to identify the model(s) within a set of alternatives that provide the best trade-off(s) between likelihood and complexity. However, the AIC penalises all model parameters equally, which may not be appropriate when they differ in their ability to influence a fit. In this study, we use the Leave-one-out information criterion (LOO-IC; Vehtari et al. (2017)) which penalizes the addition of model parameters only to the extent that they are informed by the data and so can contribute to overfitting.

Briefly, we define the log predictive density of a single observation y_i given a model with parameters θ – that is, the average value of the log-likelihood $\log\big(p(y_i|\theta)\big)$ across the joint posterior distribution of θ. We approximate this by making D draws from the posterior distribution, calculating the likelihood of y_i for each set of parameters, and averaging. This process is repeated for each data point $(y_1, ..., y_n)$ to calculate the log point-wise predictive density (lppd) for the whole dataset:

$$\text{lppd} = \sum_{i=1}^{n} \log\left(\frac{1}{D}\sum_{d=1}^{D} p(y_i|\theta^{d}_{\text{post}})\right). \tag{S10}$$

One then uses the leave-one-out (*loo*) method, a special case of cross-validation, whereby the dataset of n observations $(y_1, ..., y_n)$ is partitioned into n training datasets each of size $n - 1$. Fitting the model to the training sample that excludes datapoint i gives a joint posterior $\theta^{(\text{-}i)}_{\text{post}}$. This posterior is then used to estimate the prediction accuracy of the model for the excluded observation i (the test sample), which is defined as the log of the average likelihood of the test sample across the posterior distribution. This likelihood is approximated by averaging over D draws from the posterior. This process is repeated, making each observation in the dataset $(y_1, ..., y_n)$ the test sample, and the lppd^{loo} is defined to be the sum of the log likelihoods of all these prediction accuracies:

$$\text{lppd}^{loo} = \sum_{i=1}^{n} \log\left(\frac{1}{D}\sum_{d=1}^{D} p(y_i|\theta^{(\text{-}i)d}_{\text{post}})\right) \tag{S11}$$

where the term in large parentheses characterizes the D posterior simulations fitted on $n-1$ observations when the ith observation is left out. The information criterion LOO-IC is defined as $-2 \times$ lppd (Vehtari et al., 2017). To calculate it we use the *loo-2.0* package in the *rstan* library, which estimates the lppd^{loo} using Pareto-smoothed importance sampling (PSIS) – an approximation of leave-one-out cross-validation that uses existing posterior draws from the model fits (Vehtari et al., 2015).

We then used the estimated LOO-IC values to assess the relative support for models using the analog of the Akaike weight – the probability that a given model will explain new data better than other models considered in the analysis. Following Burnham and Anderson (Burnham and Anderson, 2002), these weights are

$$w_i = \frac{\exp(-\frac{1}{2}\Delta\text{LOO-IC}_i)}{\sum_{m=1}^{M} \exp(\frac{1}{2}\Delta\text{LOO-IC}_m)}, \tag{S12}$$

where ΔLOO-IC$_i$ is the difference in LOO-IC values between model i of M candidates and the model with the lowest LOO-IC value.

E – Modelling the development of FM B cells in young mice

Empirical description of T1 precursor numbers in young mice

To capture the dynamics of T1 cells in young mice (Fig. 5A) we used the empirical function $S(t) = S_0\,(1 + t^n\,\exp(-bt))$, and fitted this to the log-transformed cell counts, using least squares to estimate S_0, n and b.

Explaining the developmental dynamics of FM B cells in young mice

To test the hypothesis of lower recruitment of T1 B cells in neonates than in adults, we allowed the rate of influx to increase with time early in life, approaching the value ψ estimated from our best-fitting model in adults aged 7 weeks and older; we assumed the form $\psi(t) = \psi(1 - \exp(-r_\psi t))$. The estimated rate r_ψ was sufficiently large that $\psi(t)$ was very close to ψ at age 7 weeks (Fig. 5E).

To test whether FM B cells in young mice are lost more rapidly than those in adult mice, we extended the time-dependent loss model, in which we had described the loss rate from age t_0=7 weeks onwards as $\delta(t) = \delta_0 e^{-r(t-t_0)}$. For $t < t_0$ we assumed $\delta(t) = \delta_0(1 - e^{r_n(t-t_0)})$ with $r_n > r$ (Fig. 5F).

We fitted both extensions of the time-dependent loss model to the counts of FM B cells in young mice separately, estimating r_ψ and r_n in the process.

Estimating the age distribution of FM B cells in young mice

To generate the predicted age distributions of cells under the two models above, we recast the models as partial differential equations (PDEs) that explicitly track cell age. In the time-dependent loss model, the population density of FM B cells of age a in mice of age t is given by the solution to

$$\frac{\partial N(a,t)}{\partial a} + \frac{\partial N(a,t)}{\partial t} = -(\delta(t) - \alpha)\,N(a,t). \tag{S13}$$

The rate of influx of cells of age zero $N(0,t)$ is the source influx $\psi S(t)$, or $\psi(t)S(t)$ for the model in which the *per capita* influx rate varies with age. The other boundary condition is the age distribution of cells at time zero, size $N(a,0)$. We assumed that the FM B cell compartment at the time of birth is sufficiently small that we could set $N(a,0) = 0$. We solved this model using the parameters estimated from fitting the extensions of time-dependent loss model (described above) to the total counts of FM B cells in young mice. We then calculated the normalised

cell age distribution of FM B cells at $t = 7$ weeks using

$$G(a,t) = \frac{N(a,t)}{\int_0^t N(a,t)da}.$$

See Rane et al. (2018) for full details of the solution of this class of model.

Modelling the distribution of GFP expression in FM B cells

We assumed that the GFP intensity within FM B cells declines exponentially with their age, since there is no residual Rag activity from the T1 stage onwards (Yu et al., 1999) and the estimated slow rate of self-renewal of FM B cells means that dilution of GFP through division is minimal. Newly matured FM B cells of age $a = 0$ therefore have GFP intensity $f_{\max}$, which then declines as $f(a) = f_{\max} e^{-\gamma a}$, and $f_{\max}$ and γ are parameters to be estimated. There is therefore a 1-to-1 correspondence between GFP expression f and cell age a, and so we recast the FM B cell population density $N(a,t)$ (the solution to eqn. S13) as

$$\begin{aligned} N(f,t) &= N(a,t)\left|\frac{da}{df}\right| \\ &= N\left(\frac{\ln f_{\max} - \ln f}{\gamma}, t\right)\frac{1}{\gamma f} \end{aligned} \tag{S14}$$

where the Jacobian da/df preserves local cell densities under coordinate transformations. The mean fluorescence intensity (MFI) of GFP in FM B cells in a mouse of age t is then

$$\text{MFI}(t) = \frac{\int_{f_{\min}}^{f_{\max}} f\, N(f,t)\, df}{\int_{f_{\min}}^{f_{\max}} N(f,t)\, df}, \tag{S15}$$

where $f_{\min}$ is a lower cutoff in GFP expression needed to avoid divergence in the integral (GFP never decays to zero) and was set to 0.1, well below all values observed experimentally. For each model, we estimated $f_{\max}$ and γ by fitting eqn. S15 to the observed timecourse of the MFI of GFP in FM B cells. Using the solution $N(f,t)$ for each model we then predicted the timecourse of the MFI within GFP^{pos} FM B cells only, using a gating threshold of $f_{\min} = 1000$.

F – Using information from Ki67-reporter mice to aid discrimination between models of GC B cell dynamics

The Ki67-Cre-ER^{T2}-YFP system allows us to track cohorts of cells that underwent cell division during tamoxifen treatment. We measured the frequencies of YFP-expressing cells at day 4 and day 62 post-tamoxifen and, for each model (as described below), used the decline in YFP expression over this time period to constrain the rates of loss and/or division. YFP expression is preserved upon cell division but is diluted by loss or onward differentiation. To illustrate, for the simple homogeneous model, YFP expression will decline at the net rate of loss of the population λ, which is $\delta - \alpha$. We can therefore relate λ to the fold loss of YFP expression over a time τ:

$$Y(t) = Y_0\, e^{-\lambda \tau} \implies \lambda = -\log(\Delta Y)/\tau. \tag{S16}$$

Priors on ΔY. We took the strategy of making ΔY a parameter in the model, using its observed values to generate its prior; and sampling from this prior then allowed us to estimate or constrain other parameters. For splenic GC B cells, the mean YFP-labelled fraction dropped from 0.35 to 0.04 over 8 weeks, yielding $\Delta Y \sim 0.12$. This, together with the scatter in ΔY observed in YFP reporter mice (4 mice at the 2 week timepoint and 5 mice at the 8 week timepoint, unpaired data; Fig. 6D in the text), suggested $\Delta Y \sim \mathcal{N}(0.12, 0.05)$. Lymph node GC B cells exhibited

$\Delta Y \simeq 0.42$. When assuming T1 or T2 as their precursors, which turn over rapidly and are therefore not expected to provide a persistent source of YFP-labelled cells after withdrawal of tamoxifen, we therefore assumed $\Delta Y \sim \mathcal{N}(0.42, 0.05)$. When assuming FM B cells to be precursors, which turn over more slowly, we considered the possibility that FM B cells might act as a reservoir that feeds new YFP$^+$ cells into LNGC for some time after withdrawal of tamoxifen. In the case the drop in YFP expression yields only a lower bound on λ. Accordingly, we assumed that ΔY was skew-normally distributed with a bias towards values less than the mean of 0.42, and a standard deviation of 0.1 ($\Delta Y \sim$ SkewNormal$(0.42, 0.1, -5)$). Specifically, if $\mu \in \mathbb{R}, \sigma \in \mathbb{R}^+$, and $k \in \mathbb{R}$, then for y $\in \mathbb{R}$,

$$\text{SkewNormal}(y \mid \mu, \sigma, k) = \frac{1}{\sigma\sqrt{2\pi}}\, e^{-\frac{(y-\mu)^2}{2\sigma^2}} \left(1 + \text{erf}\left(k(\frac{y-\mu}{\sigma\sqrt{2}})\right)\right),$$

where 'erf' is the Gaussian error function. For each model we used the YFP information in the following ways:

Simple homogeneous model (with or without time-dependent influx): Using the above priors on ΔY and the division rate α, we then estimated the rate of loss (δ) using equation S16;

$$\delta = \frac{-\log(\Delta Y)}{\tau} + \alpha.$$

Time-dependent division or loss: With time dependent division, we assumed priors for ΔY and δ and calculated the rate of division at t_0, which we denote α_0. With time dependent loss loss, we assumed priors for ΔY and α and calculated the rate of loss at t_0 (δ_0):

$$\begin{aligned} \alpha_0 &= \frac{\log(\Delta Y) + \delta\tau}{\tau\, e^{\tau\, r}} \\ \delta_0 &= \frac{-\log(\Delta Y) + \alpha\tau}{\tau\, e^{\tau\, r}}. \end{aligned} \tag{S17}$$

Kinetic heterogeneity: This model (eqns. S3) predicts a biphasic loss of YFP, reflecting the net loss rates of the transient (λ_f) and persistent (λ_s) subsets and which were present at unknown frequencies q and $1-q$, respectively. We then assigned priors ΔY, λ_f and λ_s, to give q:

$$\begin{aligned} Y(\tau) &= Y_0^f\, e^{-\lambda_f\,\tau} + Y_0^s\, e^{-\lambda_s\,\tau} \\ \Delta Y = Y(\tau)/Y(0) &= q\, e^{-\lambda_f\,\tau} + (1-q)\, e^{-\lambda_s\,\tau} \\ \implies q &= \frac{\Delta Y - e^{-\lambda_s\,\tau}}{e^{-\lambda_f\,\tau} - e^{-\lambda_s\,\tau}}. \end{aligned} \tag{S18}$$

Here Y_0^f and Y_0^s are the fractions of YFP-labelled cells in the transient and persistent subsets, respectively, at time $\tau = 0$. Assuming $\lambda_f > \lambda_s$, the constraint $0 < q < 1$ in turn constrains the priors on λ_f and λ_s;

$$\lambda_s < \frac{-\log(\Delta Y)}{\tau} < \lambda_f.$$

Placing priors on the division rates α_f and α_s, we could then calculate the rates of loss of transient (δ_f) and persistent subsets (δ_s) using

$$\begin{aligned} \delta_f &= \lambda_f + \alpha_f \\ \delta_s &= \lambda_s + \alpha_s. \end{aligned}$$

Incumbent: We derived a similar relationship between $\lambda_{\text{displaceable}}$ and $\lambda_{\text{incumbent}}$ to that in eqn. S18,

$$\lambda_{\text{incumbent}} < \frac{-\log(\Delta Y)}{\tau} < \lambda_{\text{displaceable}}.$$

Using priors on ΔY, $\lambda_{\text{displaceable}}$, $\lambda_{\text{incumbent}}$, α_{inc} and α, we calculated δ and δ_{inc}:

$$\delta = \lambda_{\text{displaceable}} + \alpha$$
$$\delta_{\text{inc}} = \lambda_{\text{incumbent}} + \alpha_{\text{inc}}.$$
